# The microbiologist's guide to metaproteomics

**DOI:** 10.1002/imt2.70031

**Published:** 2025-05-06

**Authors:** Tim Van Den Bossche, Jean Armengaud, Dirk Benndorf, Jose Alfredo Blakeley‐Ruiz, Madita Brauer, Kai Cheng, Marybeth Creskey, Daniel Figeys, Lucia Grenga, Timothy J. Griffin, Céline Henry, Robert L. Hettich, Tanja Holstein, Pratik D. Jagtap, Nico Jehmlich, Jonghyun Kim, Manuel Kleiner, Benoit J. Kunath, Xuxa Malliet, Lennart Martens, Subina Mehta, Bart Mesuere, Zhibin Ning, Alessandro Tanca, Sergio Uzzau, Pieter Verschaffelt, Jing Wang, Paul Wilmes, Xu Zhang, Xin Zhang, Leyuan Li, The Metaproteomics Initiative

**Affiliations:** ^1^ Department of Biomolecular Medicine, Faculty of Medicine and Health Sciences Ghent University Ghent Belgium; ^2^ VIB‐UGent Center for Medical Biotechnology, VIB Ghent Belgium; ^3^ Département Médicaments Et Technologies Pour La Santé (DMTS) Université Paris‐Saclay, CEA, INRAE, SPI, Bagnols‐Sur‐Cèze France; ^4^ Applied Biosciences and Process Engineering Anhalt University of Applied Sciences Köthen Germany; ^5^ Bioprocess Engineering, Max Planck Institute for Dynamics of Complex Technical Systems Magdeburg Germany; ^6^ Bioprocess Engineering Otto von Guericke University Magdeburg Germany; ^7^ Department of Plant and Microbial Biology North Carolina State University Raleigh North Carolina USA; ^8^ Molecular Disease Mechanisms Group, Department of Life Sciences and Medicine University of Luxembourg Esch‐sur‐Alzette Luxembourg; ^9^ Institute for Advanced Studies University of Luxembourg Esch‐sur‐Alzette Luxembourg; ^10^ School of Pharmaceutical Sciences, Faculty of Medicine University of Ottawa Ottawa Ontario Canada; ^11^ Biologic and Radiopharmaceutical Drugs Directorate, Health Products and Food Branch Health Canada Ottawa Ontario Canada; ^12^ Quadram Institute Bioscience, Norwich Research Park Norwich Norfolk UK; ^13^ University of East Anglia Norwich Norfolk UK; ^14^ Department of Biochemistry, Molecular Biology, and Biophysics University of Minnesota, Minneapolis Minnesota USA; ^15^ Université Paris‐Saclay, INRAE, AgroParisTech, Micalis Institute PAPPSO Jouy‐en‐Josas France; ^16^ Biosciences Division Oak Ridge National Laboratory Oak Ridge Tennessee USA; ^17^ Data Competence Center MF2, Robert‐Koch‐Institut Berlin Germany; ^18^ Department of Molecular Toxicology Helmholtz‐Centre for Environmental Research ‐ UFZ GmbH Leipzig Germany; ^19^ Luxembourg Centre for Systems Biomedicine University of Luxembourg Esch‐sur‐Alzette Luxembourg; ^20^ BioOrganic Mass Spectrometry Laboratory (LSMBO), IPHC UMR 7178 University of Strasbourg, CNRS Strasbourg France; ^21^ Department of Mathematics, Statistics and Computer Science, Faculty of Sciences Ghent University Ghent Belgium; ^22^ Department of Biomedical Sciences University of Sassari Sassari Italy; ^23^ Unit of Microbiology and Virology, University Hospital of Sassari Sassari Italy; ^24^ State Key Laboratory of Medical Proteomics, Beijing Proteome Research Center, National Center for Protein Sciences (Beijing) Beijing Institute of Lifeomics Beijing China; ^25^ Department of Life Sciences and Medicine, Faculty of Science, Technology and Medicine University of Luxembourg Belvaux Luxembourg

**Keywords:** bioinformatics, functional dynamics, mass spectrometry, metaproteomics, microbiome

## Abstract

Metaproteomics is an emerging approach for studying microbiomes, offering the ability to characterize proteins that underpin microbial functionality within diverse ecosystems. As the primary catalytic and structural components of microbiomes, proteins provide unique insights into the active processes and ecological roles of microbial communities. By integrating metaproteomics with other omics disciplines, researchers can gain a comprehensive understanding of microbial ecology, interactions, and functional dynamics. This review, developed by the Metaproteomics Initiative (www.metaproteomics.org), serves as a practical guide for both microbiome and proteomics researchers, presenting key principles, state‐of‐the‐art methodologies, and analytical workflows essential to metaproteomics. Topics covered include experimental design, sample preparation, mass spectrometry techniques, data analysis strategies, and statistical approaches.

## Highlights

- Metaproteomics provides a direct functional perspective on microbiome dynamics, offering unique insights that complement other multi‐omics approaches.
- This guide offers accessible, up‐to‐date methodologies for experimental design, sample preparation, mass spectrometry, and data analysis in metaproteomics.
- Developed by the Metaproteomics Initiative (www.metaproteomics.org), this guide is part of our ongoing efforts to educate researchers about metaproteomics, address technical challenges, and foster collaborations to advance microbiome research.

## Graphical abstract

Metaproteomics within the multi‐omics framework provides a comprehensive view of microbial systems by identifying proteins, quantifying their levels, detecting posttranslational modifications (PTMs), mapping protein–protein interactions (PPIs), and localizing proteins. It complements other omics techniques, such as metagenomics, metatranscriptomics, and metabolomics, to deepen our understanding of microbiome dynamics across various research domains.

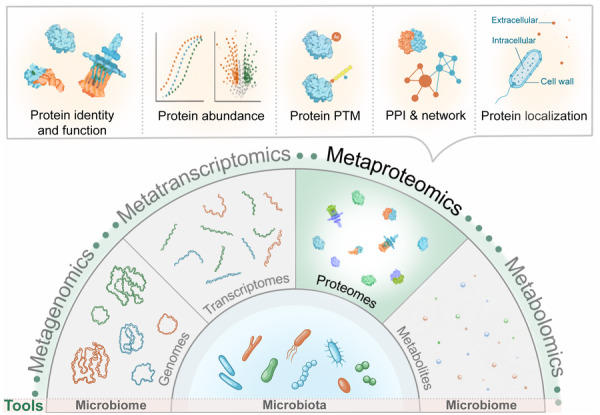

## INTRODUCTION

The importance of microbiomes in nearly all processes within the biosphere is increasingly clear. Composed of bacteria, bacteriophages, archaea, yeasts, fungi, protozoa, and viruses, microbiomes are highly diverse in taxonomic composition. A microbiome and its theater of activity—including microbial elements such as genes, transcripts, proteins, and metabolites—together form a microbiome [1]. Microbiomes are, in most cases, highly structured in both membership and function. This underscores the need to understand microbiomes and their interactions with their environment or eukaryotic hosts, whether beneficial or harmful. However, the complexity of these systems challenges traditional research tools, particularly cultivation‐dependent approaches, which, given the wealth of intra‐organism interactions, are not scalable for large‐scale microbiome studies.

The rapid advancement of omics‐based approaches has opened new avenues for systems biology‐based research into the complexity of microbiomes. Shotgun metagenomics, in particular, has proven to be a powerful tool, offering much deeper insights than older techniques such as 16S rRNA gene amplicon sequencing. Metagenomics enables the discovery of complete genomic inventories, even for uncultured microorganisms, revealing the metabolic and physiological capabilities of a microbiome. However, it is limited to predicting functions rather than identifying active processes. To overcome this limitation, omics approaches such as metatranscriptomics, metaproteomics, and metabolomics provide essential insights into actual gene expression and activity under specific conditions. Together, these techniques bridge the gap from taxonomic structure to genomic potential and dynamic, context‐dependent functions.

**Figure 1:**
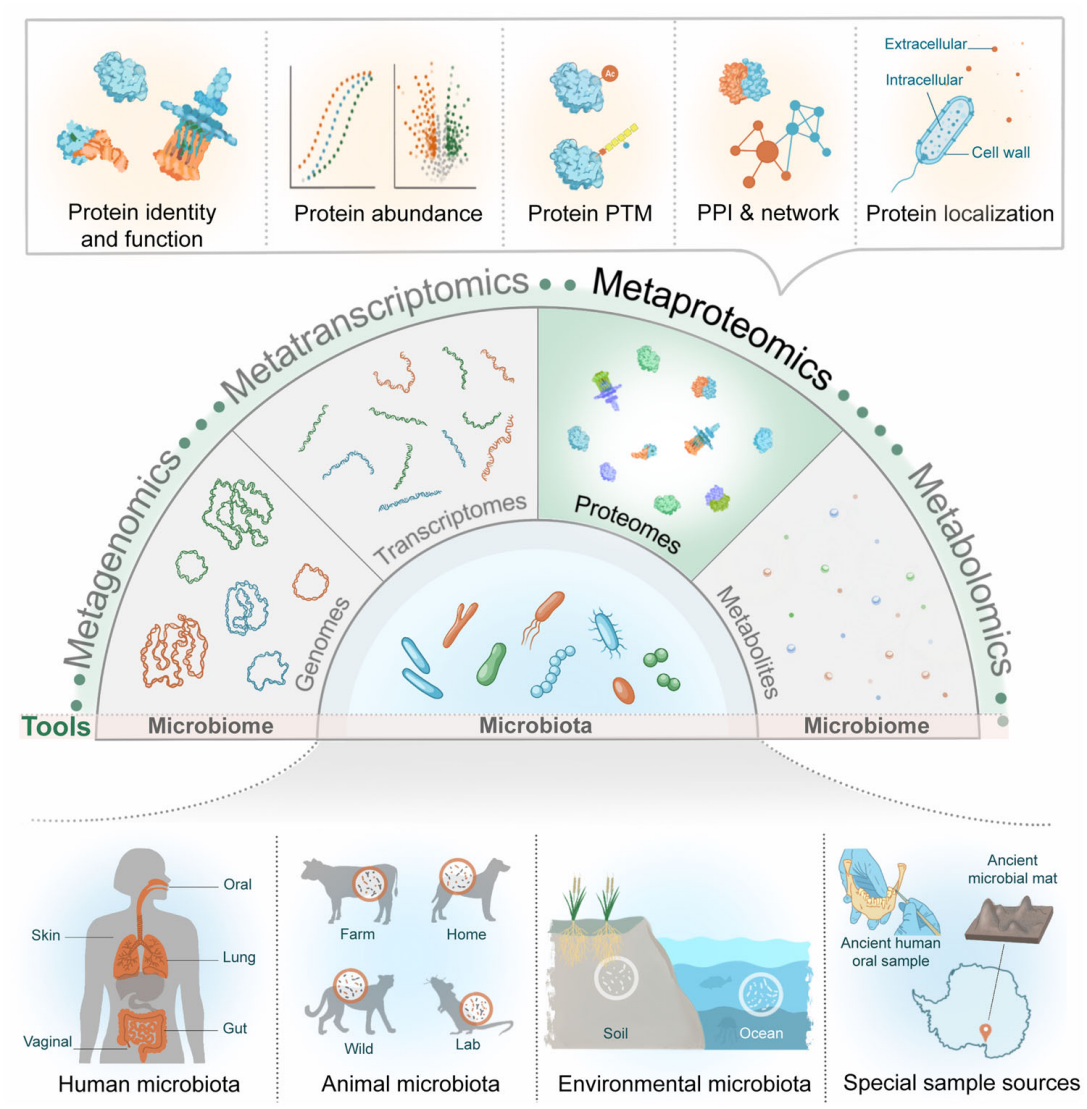
Overview of metaproteomics within the multi‐meta‐omics toolbox applied to diverse microbiome research domains. This figure highlights the role of metaproteomics in identifying proteins, quantifying their abundances, detecting posttranslational modifications (PTMs), mapping protein–protein interactions (PPIs), and determining protein localizations. Metaproteomics complements other omics approaches, including metagenomics, metatranscriptomics, and metabolomics, to provide a comprehensive understanding of microbial systems. Examples of microbiome research domains include the human microbiome (oral, skin, gut, lung, and vaginal), animal microbiomes (farm, wild, and laboratory animals), environmental microbiomes (soil and ocean), and special sample sources (e.g., ancient microbiome samples).

Metaproteomics enables the comprehensive analysis of the proteins expressed and functional in a microbiome, quantifies their abundances, and characterizes their modifications, interactions, and localizations (Figure 1). Proteins serve as the primary catalytic units and structural elements of microbiomes, making metaproteomics a direct reflection of the microbiome's phenotype [2]. This approach provides a detailed functional description and examines specific protein changes associated with structure, homeostasis, and enzymatic activity. Differences in protein sequences allow researchers to determine the taxonomic origins of particular enzyme sets, linking functions to taxonomic units.

Metaproteomics has already been successfully applied in the context of many impactful studies. It has contributed to fundamental understanding of microbial ecology, host‐microorganism interactions, and disease mechanisms [3]. It has also improved biotechnological processes such as anaerobic digestion and wastewater treatment [4, 5, 6], supported environmental monitoring [7], and improved agricultural productivity [8, 9]. Furthermore, it has applications in describing historical heritage and solving forensic questions [10]. Readers interested in further details on the benefits of metaproteomics can explore several recommended reviews [11, 12, 13, 14, 15] and perspectives on its future [16, 17, 18, 19].

Below, we will discuss three applications in more depth, starting with deciphering microbial activity in the ocean for bio‐monitoring. The ocean plays an important role in global climate regulation, carbon storage, and environmental pollution. Understanding aquatic carbon and nitrogen fixation processes under both steady‐state and changing environmental conditions, as well as pollutant degradation, is essential for improving climate change and pollution monitoring. Recent metaproteomic studies have shed light on the role of micro‐nutrients in regulating ammonium and carbon processes and have helped decipher biogeochemical processes across scales [20, 21, 22]. Importantly, the work allowed us to map nitrification and carbon metabolism processes in different regions and depths and to identify micro‐nutrient limitation, particularly zinc limitation indicated by zinc responsive proteins, as a key modulator of microbial and algal activity in the ocean [20, 21]. At the same time, metaproteomics has enabled scientists to decipher the different roles of zooplankton, bacteria, archaea, and viruses in carbon cycling in the deep sea. Among other findings, this study suggests that the high abundance of extracellular enzymes of comparatively low abundant gammaproteobacteria in the deep sea, which might be driven by bacteriophages‐induced cell lysis, promotes carbon cycle under hydrostatic pressure [22]. Together, these insights have important implications for monitoring ocean activity and may enable the use of microbial scavenger proteins for bio‐monitoring, such as tracking local zinc levels.

The second application focuses on optimizing biofuel production and feeding efficiency using metaproteomics. The growing demand for agricultural and biotechnological products, including vegetables, meat, and fuel, combined with increasing waste production such as plastic and wastewater, call for more efficient production and waste management strategies. Metaproteomics has contributed to deciphering microbial pathways for plastic degradation in the ocean, leading to the identification of polyamidase, hydrolase, and depolymerase, that is, enzymes synthesized by rare taxa [23]. These taxa and enzymes hold promise for large‐scale industrial scale plastic degradation. Similarly, metaproteomics helped our understanding of microbial players and specific carbohydrate‐active enzymes in lignocellulose biofuel production. This knowledge now enables the optimization of biofuel production at high solids loads, a critical factor for industrial‐scale efficiency [24]. Microbiome composition and carbohydrate‐active enzymes also play an important role in cattle feed efficiency. However, only a recent metaproteomics‐based analysis of rumen microbiota from different cows has demonstrated that functional redundancy on protein level and niche partitioning are the underlying factors influencing feed efficiencies [25]. These findings provide a foundation for developing pre‐ and pro‐biotic intervention strategies to optimize feeding efficiency in response to limited resources and increasing demand.

The third application focuses on identifying biomedical disease markers. The human microbiome has received substantial attention for its role in disease initiation, progression, and therapy resistance. While dysbiotic community profiles have been characterized for various disease states, functional redundancy among different microbial community patterns limits their diagnostic utility, necessitating the use of other omics techniques. Metaproteomics has recently provided deeper insights into the complex interactions between diet, the host, and the microbiome in inflammatory bowel disease (IBD) patients. This approach has led to the identification of novel biomarkers that may outperform calprotectin as an inflammation marker. Notably, these are biomarkers that could not be detected at the taxonomic level. The study also established a link between fecal dietary protein, malabsorption in the small intestine, and inflammation [26]. These findings highlight the advantages of metaproteomics over other techniques for studying diet‐related diseases and dietary interventions, as it uniquely enables the simultaneous analysis of microbiome composition, host responses, and dietary components.

In addition, the strength of metaproteomics in these application fields lies in its ability to address many important questions, such as: (i) What are the metabolic and physiological processes of microorganisms in diverse habitats, including environmental, technical, and host‐associated systems? (ii) How do microbiomes respond to changing conditions, as reflected by differential protein expression? (iii) How do microbes interact with their environment, including extracellular and intracellular protein dynamics? (iv) What posttranslational modifications (PTMs) regulate protein activity and structure? (v) How do microbiome phenotypes change over time or across spatial scales? (vi) How can stable isotope information from metaproteomes represent microbial activity and substrate utilization [4, 27]?

This review, prepared by the Metaproteomics Initiative (www.metaproteomics.org), aims to serve as a practical and accessible guide to metaproteomics. A detailed overview of the organization and presentation of this collaborative work is provided in Section A collaborative effort: Writing a comprehensive review with members of the Metaproteomics Initiative, highlighting our dedication to delivering a comprehensive and valuable resource for the microbiome research community.

## BASICS OF PROTEOMICS

Proteins are the essential structures and machinery that execute the instructions encoded in DNA, performing tasks ranging from catalyzing biochemical reactions to providing structural support. The term “proteome” refers to the complete set of proteins expressed in a cell, tissue, or organism [28]. Proteomics, as a field, seeks to uncover the identities, quantities, structures, interactions, and modifications of proteins to better understand their roles in biological systems.

Although the term “proteome” was coined in the mid‐1990s, its foundations lie in decades of protein biochemistry research that continues to shape modern proteomics. One of the earliest applications of proteomics combined gel electrophoresis (1D and 2D) with mass spectrometry techniques such as matrix‐assisted laser desorption‐ionization (MALDI) and electrospray ionization‐liquid chromatography‐tandem mass spectrometry (ESI‐LC‐MS/MS) [29]. Initially, protein samples were separated on a combination of 1D and 2D gels. One gel was electro‐blotted onto a nitrocellulose membrane and stained using amido black, while the other gel was silver‐stained for higher sensitivity. Protein bands or spots were excised from the nitrocellulose membrane, digested with trypsin, and identified using mass spectrometry. Aligning the nitrocellulose membrane with the silver‐stained gel allowed researchers to locate bands that were difficult to visualize on the less‐sensitive stain. Subsequent improvements, such as in‐gel digestion, eliminated the need for electro‐blotting. Early proteomics efforts also gave rise to software tools that automated protein identification, and therefore replaced manual annotation of peptide sequences. Many of these early innovations, however, formed the basis for modern proteomics workflows.

The development of gel‐free proteomics marked a significant advancement in the field. This approach bypasses gel‐based separation, proceeding directly from protein extraction to digestion and mass spectrometry. Gel‐free methods catalyzed a wave of new techniques, reagents (e.g., stable isotope labeling by amino acids in cell culture [SILAC], isotope‐coded affinity tags [ICAT], isobaric tags for relative and absolute quantification [iTRAQ]), and software, which collectively improved protein identification, posttranslational modification (PTM) analysis, quantitation, and multiplexing. Tasks that were once labor‐intensive with 2D gel mass spectrometry (MS) became faster and more accessible through gel‐free workflows. Moreover, mass spectrometers, which were initially optimized for small molecule research, were adapted for proteomics. Over the past 15 years, proteomics‐dedicated mass spectrometers have been developed, offering greater speed, sensitivity, and accuracy in peptide identification and quantitation.

Proteomics today falls into two broad methodological categories: shotgun (or bottom‐up) proteomics [30] and top‐down proteomics [31]. Shotgun proteomics, the more widely used approach, involves enzymatic digestion of proteins into peptides, which are analyzed by mass spectrometry. This method is robust and effective for protein identification and quantification. In contrast, top‐down proteomics directly analyzes intact proteins, providing insights into sequences, structures, and modifications. Although top‐down proteomics offers unique advantages, it is technically demanding, less commonly used in single‐species proteomics, and not currently applied in metaproteomics.

A typical bottom‐up proteomics workflow begins with the enzymatic digestion of proteins, most commonly using trypsin, into smaller peptides. These peptides are separated through liquid chromatography and analyzed by tandem mass spectrometry (LC‐MS/MS). In the mass spectrometer, the peptides are ionized, and their intact forms are detected to generate MS1 spectra. The peptides are further fragmented to produce MS2 spectra, which are analyzed by proteomics software. In most cases, database searches match these spectra to theoretical spectra derived from protein databases. This approach enables the identification and quantification of peptides and their corresponding proteins. For those seeking a deeper understanding of proteomics, numerous resources and reviews provide detailed insights into the field [32, 33, 34, 35].

## EXPERIMENTAL METHODS IN METAPROTEOMICS

Metaproteomics expands upon proteomics techniques, leveraging high‐resolution LC‐MS/MS instruments [36, 37] and accompanying software tools for mass spectra identification. However, metaproteomics goes beyond the straightforward application of proteomics to microbiome research. Its added complexity arises from the requirement to consider both species‐specific and functional annotations for each protein. Additionally, the presence of protein homologs across phylogenetically related species within a single sample further complicates protein inference.

The key distinctions between proteomics and metaproteomics lie in the taxonomic and functional complexity of microbiomes, the vast size of microbiome databases, and the challenges associated with sample processing, as well as the identification and quantitation of peptides and proteins. Additionally, specialized bioinformatic and statistical tools are required to track both the taxonomic and functional annotations of peptides and proteins. These aspects, which are unique to metaproteomics, will be discussed in detail throughout the remainder of this article.

**Figure 2:**
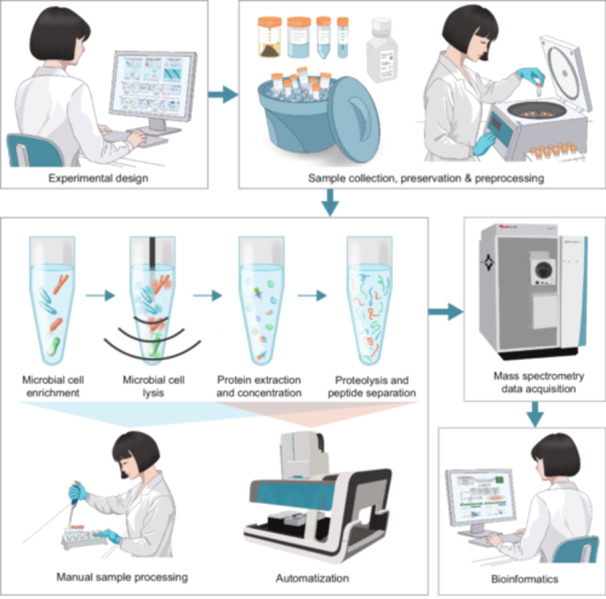
Overview of key principles and workflows in metaproteomics. A typical metaproteomics workflow begins with experimental design (Section Experiment design), followed by sample collection, preservation, and preprocessing (Sections Sample collection, preservation, and storage prior to before preprocessing to Sample preprocessing). Microbial cells undergo enrichment, lysis, protein extraction, and peptide separation, processed either manually (Sections Protein sample preparation: From extraction to digestion to Separation and fractionation techniques) or automated (Section Automation) before mass spectrometry data acquisition (Section Mass spectrometry data acquisition methods). Finally, bioinformatics analysis (Section Computational analysis of metaproteomics data) performs database searches and interprets the data to reveal microbial functions and ecological insights.

This section provides an essential foundational guide to start with metaproteomics studies (Figure 2). We outline the basic principles for each step, starting with experimental design (Section Experiment design), followed by sample collection, preservation, and preprocessing (Sections Sample collection, preservation, and storage prior to before preprocessing and Sample preprocessing). Protein sample preparation is then described, covering both manual workflows (Sections Protein sample preparation: From extraction to digestion and Separation and fractionation techniques) and automated workflows (Section Automation). Next, we explain the basics of MS data acquisition (Section Mass spectrometry data acquisition methods), before delving into the detailed bioinformatics workflows used in metaproteomics (Sections Peptide identification, protein inference, and quantification to Downstream statistics).

### Experiment design

#### Aligning experimental design with the scientific question

**Figure 3:**
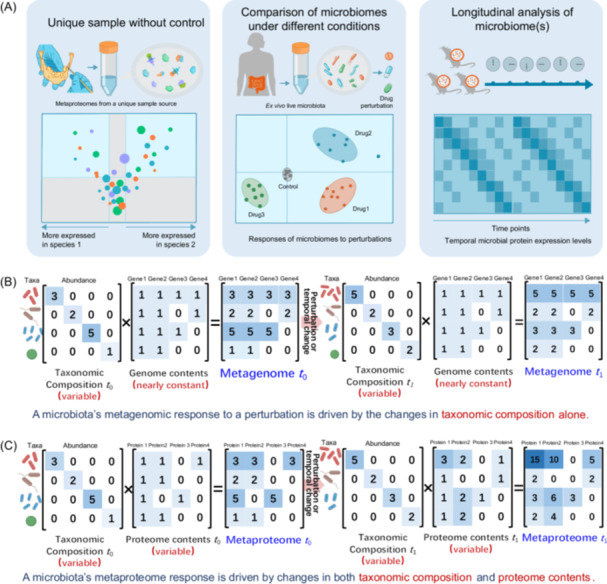
Metaproteomic experimental designs and their comparison with metagenomics in studying microbiome dynamics. (A) Overview of common metaproteomic experimental designs. The left panel illustrates the comparison of microbial protein expression between species within a unique sample source, lacking a control. The middle panel compares microbiomes under varying conditions, such as drug treatments, using ex vivo microbiomes to assess microbial responses. The right panel shows longitudinal studies that monitor temporal changes in microbial protein expression over time. (B) Metagenomic responses to perturbations, showing shifts in taxonomic composition while assuming genome content remains relatively constant. (C) Metaproteomic responses to perturbations, showing changes in both taxonomic composition and proteome content. This approach captures microbial abundances and their functional contributions, providing deeper insights into microbiome dynamics.

A well‐designed metaproteomics experiment forms the basis for generating meaningful insights that directly address the scientific question being studied. Most importantly, the experimental design must align with the specific scientific question being addressed and the resources available to answer that question. Broadly, three experimental scenarios can be outlined (Figure 3A). (i) Unique sample without a control: The goal here is to provide a comprehensive description of the taxonomic and functional units present in the sample, although comparisons with a control are not possible. Examples include desiccated material from a historical Antarctic ice core [38], a unique biofilm from an industrial storage pool [39], residues from an ancient tomb [40], or medieval dental calculus [41] were analyzed using metaproteomics. Differential functional abundances among the identified microorganisms can reveal their metabolic specialization. (ii) Comparison of microbiomes under different conditions: This common approach highlights differences between conditions. Comparisons may involve two conditions (i.e., condition A vs. condition B) or more complex setups with multiple conditions. Specific cases include dose–response analyses, where a single parameter such as stress intensity is modified, or spatial comparisons. Examples include characterizing microbial communities along a 5000 km Pacific Ocean transect [21] or analyzing microbiome responses to various xenobiotics in vitro [42]. (iii) Longitudinal analysis of a single microbiome or multiple microbiomes: This strategy captures temporal dynamics within a microbial community, and potentially the host's response, by analyzing the same microbiome at different time points. A more complex approach examines temporal changes across multiple conditions or sampling sites. Examples include monitoring gut microbiomes in Crohn's disease patients post‐resection surgery over 1 year [43] or monthly analyses of specialized microbiomes in a two‐stage anaerobic digester for lignocellulose breakdown, tracking the dynamics between hydrolytic and methanogenic subsystems [44].

Some readers may already have experience designing experiments for metagenomics and understand its principles. In contrast, metaproteomics offers a different perspective on microbiome changes (Figure 3B,C). Metagenomics captures shifts driven by changes in taxonomic composition, as genomic content within a sample is relatively constant (Figure 3B). This approach reveals species abundance and diversity but does not provide functional insights. Metaproteomics, on the other hand, measures not only taxonomic changes through taxon‐specific peptide intensities but also dynamic functional responses through proteome variations across taxa (Figure 3C). This makes metaproteomics particularly well‐suited for comparing microbiomes under different conditions or for longitudinal studies.

When selecting conditions or time points for a kinetic analysis, careful consideration is essential. Comparisons between vastly different samples, such as a soil microbiome versus a human gut microbiome, are in general uninformative, while overly similar samples may show no significant differences. Selection should be guided by a clear rationale and preliminary observations. The reference condition or time point depends on the scientific question but may involve using a mixture of all samples as a reference. While this approach increases peptide diversity in the reference sample, it can complicate analysis if the full diversity is not captured by the analytical workflow as further detailed in Sections Separation and fractionation techniques and Mass spectrometry data acquisition methods.

Potential confounding factors must also be accounted for during experimental design [45]. Comprehensive metadata collection is critical, including information on sampling location, timing, storage, processing conditions, and data acquisition. Additional metadata, such as weather conditions on sampling days, patient medication, or health status, may also be essential for interpreting results. Additionally, researchers should also consider using additional material to create appropriate databases for matching spectra to peptides and for testing methodologies before processing all samples. More details on proteomics software and database creation are provided in Sections Peptide identification with proteomics search engines and Database construction or selection, respectively.

Finally, while a limited number of metaproteomics studies have used metabolic labeling [46, 47, 48], this approach is often impractical for environmental or human microbiome samples. Metabolic labeling, as briefly mentioned in Section Basics of proteomics, involves incorporating heavy isotopes like ^15^N or ^13^C into proteins through labeled substrates, enabling the study of metabolic crosstalk and protein production rates. However, its limited applicability means that it is not further discussed in this review.

#### Reproducibility and statistics

The high complexity and heterogeneity of metaproteomics samples necessitate careful consideration of statistical power and steps to ensure reproducibility during experimental design. Biological, technical, and analytical replicates are key to producing reliable data and accurate interpretations. Increasing the number of biological replicates improves the ability to detect smaller differences, even in the presence of high variability. When only slight differences between conditions are expected, the use of pooled samples may also be considered. Technical and analytical replicates are necessary to account for noise introduced during measurement. It is advisable to first evaluate the variability of sample preparation and the analytical workflow using a representative sample. Additionally, randomizing the order of samples before LC‐MS/MS analysis reduces the risk of bias due to the sequence in which they are processed [49]. For cases where specific sources of variability, such as batch effects, are known, blocked randomization is preferable to further minimize bias. Rigorous quality control (QC) is essential during the LC‐MS/MS phase of the metaproteomics workflow to ensure data reliability and consistency. Section Quality control of LC‐MS/MS provides further details on these QC procedures.

Determining the appropriate number of biological replicates is essential to detect meaningful biological differences, such as variations in taxon biomasses, protein abundances, or metabolic pathways. Power analysis is typically used to calculate the required sample size, but it can be challenging in metaproteomics due to the complexity of experimental designs and the inherent variability of samples. When precise endpoints are unavailable, rough estimates from similar studies can serve as a guide. Power analysis considers several key factors: the effect size, which reflects the expected magnitude of differences between groups and helps determine the necessary sample size; the significance level (α), usually set at 0.05 to allow a 5% risk of false positives; statistical power (1 − β), often set at 0.8 or higher to reduce the likelihood of failing to detect a true effect; and the variability in the data, which can be estimated from pilot studies or previous literature on comparable experiments. In studies involving complex microbial communities, deriving precise sample size estimates may be impractical, but approximate estimates remain a valuable approach [50]. Conducting power analysis is critical for avoiding underpowered studies and ensuring efficient use of resources [50, 51].

### Sample collection, preservation, and storage before preprocessing

#### Sample collection and preservation

Metaproteomics has been applied to a variety of samples, including microbial communities from environmental niches such as water, soil, sewage, aerosols, and rocks [52, 53]. It has also been used to analyze microbiomes in fermented foods and beverages [54, 55] and in associations with various higher eukaryotes, including arachnids, insects, worms, mollusks, fish, plants, birds, and mammals [8, 56]. In mammals and other vertebrates, metaproteomics has been applied to numerous body sites across the digestive, respiratory, and urogenital systems [57, 58]. However, many microbiomes remain unexplored by metaproteomics.

Microbiome samples are often collected directly into sterile tubes or containers. This method is common for noninvasive clinical samples, such as feces, saliva, sputum, and urine, which can often be self‐collected by study participants [59, 60, 61, 62]. For clinical specimens requiring surface sampling, swabs, spatulas, or syringes are often used for oral, nasal, and cervicovaginal samples [63, 64, 65], while periodontal curettes or paper strips are used for tooth‐ and gingiva‐associated microbiomes [66, 67]. Invasive procedures, such as bronchoalveolar lavage, endotracheal aspiration [68], intestinal biopsies [69], colonic luminal aspirates [70], and surgical collection of colonic contents [71], are necessary for some specimens. Similarly, gastrointestinal fistulation [72] and post‐mortem dissection [73] are used for collecting samples from laboratory or field animals. For environmental samples, specialized devices such as quartz filters for bioaerosols [74] and large‐volume water transfer/filter systems for aquatic environments [75, 76] are commonly employed. More complex ecosystems may require multi‐step collection protocols [77].

The choice of collection method can significantly influence the resulting metaproteomic profile, including the ratio of microbial to nonmicrobial components and the relative abundances of microbial taxa. Collection strategies also introduce operator‐dependent variability, making user‐friendly devices especially valuable for self‐sampling of clinical specimens. In this regard, specific methodological studies are needed to investigate the impact of the different sampling protocols on the metaproteomic results [78], as well as the level of comparability between different specimens obtained to investigate the same environment or host‐related microbial community [79]. Collection strategies also introduce operator‐dependent variability, making user‐friendly devices especially valuable for self‐sampling of clinical specimens in view of their higher reproducibility. Common pitfalls, including polyethylene glycol (PEG) contamination from plasticware and keratin contamination from handling, must be carefully managed during sample collection and processing.

Microbiome sampling inherently involves the translocation of microbial communities from their native environment to laboratory conditions. During this transition, microbial communities are highly sensitive to environmental changes such as temperature, humidity, and exposure to chemical or biological agents. These factors can induce substantial alterations in the metaproteome profile. To minimize artifacts, protein extraction should ideally occur immediately after sampling. However, immediate processing is often impractical, particularly in large‐scale studies or field collections. In such cases, proper transport and storage procedures are crucial to preserving the microbiome's original biological functions. This is especially important for low‐biomass or low‐diversity microbiomes, which are more vulnerable to rapid shifts in their composition and activity due to external stimuli.

#### Storage conditions to maintain sample integrity

Proper storage is critical to preserving the integrity of microbial proteins and ensuring reliable downstream analyses. Exposure to environmental changes, such as air exposure, temperature fluctuations, or nutrient depletion, can significantly alter protein profiles, leading to misleading results. For instance, air exposure can introduce oxidative stress and enrich bacterial superoxide dismutase enzymes, which may bias colorectal cancer studies by mimicking disease‐specific characteristics [59]. Therefore, appropriate storage immediately after sample collection is essential to maintain the microbiome's original state.

The standard practice for preserving metaproteomic samples involves flash‐freezing in liquid nitrogen, followed by storage at −80°C. This approach minimizes molecular degradation and prevents alterations in protein abundance. While this method is highly effective, some experimental setups do not allow for immediate freezing. In such cases, alternative preservation methods may be employed. Solutions like PBS [80], Amies liquid medium [81], NAP buffer [82], and other commercially available liquid reagents [83] have been tested for their ability to enhance storage conditions or enable room‐temperature preservation in metaproteomics. Protease inhibitors are often added to biological fluids such as saliva to prevent uncontrolled proteolysis [84]. RNAlater or RNAlater‐like treatments have shown potential for preserving protein profiles in intestinal and marine samples, although with conflicting results [82, 85, 86]. Regardless of the method used, compatibility with downstream protein extraction, digestion, and analysis steps is crucial. The Critical Assessment of MetaProteome Investigation‐2 (CAMPI‐2) study aims to evaluate preservation protocols for efficiency and robustness by involving multiple laboratories, ensuring control over inter‐operator variability across all pre‐analytical steps. This approach allows for the identification of potential biases and the assessment of reproducibility [87].

Alternative long‐term storage strategies, such as freeze‐drying or storing samples at −20°C, in liquid nitrogen tanks, or as lyophilized powders, also require careful evaluation. These approaches may be suitable for some sample types but may not consistently maintain protein integrity. For example, frozen intact stool material has been shown to be more stable than extracted proteins when stored at −80°C, underscoring the importance of selecting storage strategies tailored to the specific sample type [88].

It is important to note that the stability of proteins during storage is highly dependent on the sample type and storage conditions. For example, the activity and stability of soil proteins are influenced by temperature, duration of storage, and soil organic matter content [89, 90]. For studies involving prolonged transport or storage, incorporating a straightforward mock community can provide valuable controls to assess sample stability and detect potential storage‐induced changes [53].

### Sample preprocessing

Sample preprocessing ensures the removal of contaminants and debris, which can hinder protein extraction, degrade analytical quality [91], and dilute biologically relevant signals. This step, as in other gene expression measurement workflows, ensures the enrichment of microbial fractions and improves the quality of downstream analysis. Ideally, preprocessing should involve minimal, rapid, and reproducible steps. Since no standardized protocols for metaproteomics (or metagenomics) currently exist, methods must be tailored to the specific sample type and evaluated based on the study's objectives [92, 93, 94]. While the breadth of samples processed for metaproteomics remains limited, this field is rapidly evolving, and many more methods are expected to emerge.

For soil samples, humic substances derived from decomposed organic material often co‐extract with proteins, interfering with MS measurements [95, 96]. To address this, several methods have been developed to remove humic compounds while preserving protein integrity before digestion [97, 98, 99, 100]. Alternatively, filter‐aided sample preparation (FASP) can directly digest proteins within humic complexes. This method uses acidification to precipitate humic compounds and undigested proteins while peptides are extracted via centrifugation through molecular weight cut‐off filters [101].

For human gut microbiome samples, nonmicrobial proteins from host cells and food debris are often much more abundant than microbial proteins, reducing the efficiency of microbial metaproteome identification [18]. Techniques such as double filtering [102] and differential centrifugation [103] can enrich microbial cells to improve identification. However, these methods may introduce biases and depend on the study's goals [92]. For example, double filtration can remove host cells and exoproteins, while differential centrifugation may nonspecifically remove microbial cells and proteins [104, 105, 106]. Moreover, these methods are time‐consuming and may be influenced by fecal variability, such as texture, fiber, and water content. Automation technologies, including solid‐phase extraction clean‐up, have been proposed to streamline processing for large longitudinal studies, reducing variability and improving reliability [107].

In studies analyzing heterogeneous samples with high host protein content, such as viscous sputum of cystic fibrosis patients, certain plant tissues or environmental samples, a homogenization step can improve sample consistency. This step should be performed under conditions (temperature and duration) that minimize alterations to the in vivo metaproteome. Various mechanical strategies can achieve homogenization, including laboratory mills [61] and glass homogenizers [108]. The addition of protease inhibitors and DNase I to prevent protein degradation and disrupt DNA‐based aggregates may also be beneficial, yet should be carefully evaluated based on the sample type and study objective.

For clinical samples containing bacterial or viral pathogens, inactivation is required before further processing outside appropriate biosafety level (BSL) containment. Since no standardized pipeline exists for this step, protocols must be tailored to the specific pathogen and sample type. Methods such as heat inactivation in lithium dodecyl sulfate buffers [109] and metabolite, protein, and lipid extraction (MPLEx), which uses chloroform, methanol, and water (8:4:3) for simultaneous pathogen inactivation and fractionation into metabolite, protein, and lipid phases, are commonly used [110]. These approaches ensure both safety and compatibility with downstream metaproteomics workflows.

### Protein sample preparation: From extraction to digestion

Preparing protein samples from biological material involves a series of interconnected steps, each essential for obtaining high‐quality metaproteomic data. The term “protein extraction” is often used broadly to describe the entire workflow of isolating proteins from a biological sample. This process typically begins with cell lysis using extraction buffers and may also include subsequent protein clean‐up steps, such as precipitation, filtration, or other methods. In some workflows, however, protein clean‐up is treated as a distinct step, especially in protocols where extraction, clean‐up, and digestion are streamlined into a single process. This section provides an overview of the key stages in protein sample preparation: cell lysis and extraction (Section Cell lysis and protein extraction), protein clean‐up (Section Protein clean‐up: Precipitation and alternative methods), protein concentration (Section Measuring protein concentration), and protein digestion (Section Protein digestion).

#### Cell lysis and protein extraction

Cell lysis releases the proteome from microbial cells, with a variety of methods available, each with distinct advantages [111]. Mechanical disruption methods, such as direct ultrasonication, noncontact ultrasonication, and bead beating, are commonly used. Ultrasonication usually involves direct ultrasonication, where the probe is directly inserted into the sample, or noncontact ultrasonication, where the sample in a tube receives sonication energy from a cup horn through a coupling fluid. An advanced noncontact method termed adaptive focused acoustic (AFA) technique provides precise control over parameters like amplitude and duration, achieving efficient lysis while minimizing protein denaturation [112]. Bead beating, which uses zirconia or silica beads, is effective for cell disruption, with bead size modulating efficiency [113].

Chemical lysis methods use detergents such as urea buffers containing Triton X‐100 or sodium dodecyl sulfate (SDS) to disrupt microbial cell membranes, often in combination with mechanical disruption/ultrasonication [113]. Notably, when combining urea‐containing buffers with mechanical disruption or ultrasonication, one should be aware of the risk of urea‐induced carbamylation caused by sample overheating [114]. Physical methods, including freeze‐thaw cycles or high‐pressure homogenization, are also effective, with pressure settings tailored to specific sample types [115]. Since microbial cell structures vary significantly, for example between Gram‐positive bacteria, Gram‐negative bacteria, and fungi, optimizing lysis conditions is crucial to preserve protein integrity, maximize yield, and ensure unbiased protein extraction [116, 117].

Recently, some of the above approaches have been compared and found that a urea‐ and SDS‐containing lysis buffer coupled to ultrasonication yielded higher protein recovery than bead beating in microbiome samples, with minimal sample loss, though both methods achieved similar peptide and protein identifications [113]. Careful selection of lysis buffers is also critical to avoid interference with downstream MS analysis. For example, ion suppression‐inducing detergents like Tween‐20 should be avoided unless they are removed during cleanup, as in methods like suspension trapping (S‐trap) or FASP.

Table 1 compares commonly used protein sample preparation methods, summarizing their key advantages and disadvantages. The choice of lysis method depends on factors such as sample type, desired protein yield, and sensitivity of proteins to denaturation or degradation. The listed lysis methods can also be combined, for example, detergent‐containing urea lysis buffers are often coupled with ultrasonication to achieve fast and unbiased bacterial cell lysis in complex microbiome samples.

**Table 1:** Comparison of standard protein sample preparation methods. This table summarizes commonly used protein sample preparation techniques, outlining their key advantages and potential disadvantages.

Method	Description	Advantages	Disadvantages
Chemical Lysis	Disrupts cell membranes with chemicals like urea or guanidine hydrochloride.	Can unfold complex proteins.	If not removed or sufficiently diluted, it can interfere with protease activity. Risk of urea‐induced carbamylation.
Detergent Lysis	Uses detergents (e.g., SDS, Triton X‐100) to solubilize cell membranes.	Mild, preserves protein function, ideal for membrane proteins.	If a detergent is not removed or sufficiently diluted, it can interfere with protease activity.
Freeze‐Thaw Cycles	Repeatedly freezes and thaws the sample to rupture cell membranes.	Simple, no special equipment needed.	Time‐consuming, may not fully lyse cells, risk of protein degradation.
Bead beating	Physical force such as using bead beating to break cell walls.	Effective for bacterial cell lysis.	Requires specific instrument, sample loss due to contact with beads, can generate heat, risk of protein degradation.
Ultrasonication	Uses ultrasound waves to break cell membranes/walls and release proteins.	Fast, effective and can be noncontact for small samples, no need for harsh chemicals.	Can denature proteins if overused, heat generation requires sample cooling.

#### Protein clean‐up: Precipitation and alternative methods

Protein precipitation addresses the challenges of complex environmental and fecal samples by removing contaminants such as lipids, nucleic acids, and polysaccharides that can interfere with downstream MS analysis. Following microbial cell lysis, effective separation of proteins from cellular debris and contaminants is essential to ensure high protein yield and purity. Removing contaminants not only improves protein recovery but also enhances MS sensitivity, enabling more accurate and reliable protein identification.

The trichloroacetic acid (TCA)/acetone precipitation method is widely employed for this purpose. This method involves adding cold (−20°C) TCA or acetone, or both, to the protein lysate to precipitate proteins, followed by centrifugation to pellet the proteins. The pellets are then washed with cold acetone (−20°C) to remove residual contaminants and insoluble particles [118]. This approach has proven effective for high‐yield protein precipitation in diverse sample types, including marine sediment and forest soil samples, which contain complex organic matrices [119]. Similarly, acidified acetone/ethanol buffer has also been used in metaproteomics [120].

An alternative method, phenol extraction, separates proteins into the organic phase while partitioning nucleic acids into the aqueous phase. This approach is particularly beneficial for “dirty” samples, such as soil and wastewater sludge, which are rich in organic and inorganic contaminants. Phenol extraction can reduce the interference caused by contaminants, thus improving the downstream analysis of target proteins [121]. Phenol extraction also enables the simultaneous extraction of nucleic acids from the same sample, making it highly suitable for integrated omics studies, especially in microbiome research [122].

For samples with low microbial load, such as fecal samples, river sediment, or air filters, maximizing protein recovery is critical. Organic solvent systems, such as chloroform/methanol or chloroform/methanol/water mixtures, have proven effective for enhancing protein recovery and minimizing the loss of low‐abundance proteins by optimizing solvent ratios and conditions [123]. Biphasic systems, such as phenol/chloroform or Triton X‐114, can also be used to selectively partition proteins and facilitate the removal of contaminants [124].

Traditional protein precipitation methods, while effective, can be labor‐intensive and may not always completely eliminate contaminants that interfere with downstream analyses. To address these limitations, alternative methods have been developed to improve protein clean‐up and digestion efficiency. Techniques such as FASP, single‐pot, solid‐phase‐enhanced sample preparation (SP3), and suspension trapping (S‐Trap) have shown promise for processing challenging samples like human fecal protein extracts [125]. Solid‐phase alkylation, a novel strategy designed for low‐loss and anti‐interference sample preparation, utilizing covalent binding and purification of proteins, has also been proved effective for marine microbiome samples [76]. These approaches integrate clean‐up and digestion steps into a single workflow, facilitating high‐throughput applications.

#### Measuring protein concentration

Accurate protein concentration measurement ensures uniform loading in downstream LC‐MS/MS analyses and facilitates reliable data interpretation [126]. Consistent peptide loading in LC‐MS/MS is essential for accurate peptide quantification, as it maintains signal intensity and ensures reliable peptide detection across samples. Uniform loading also optimizes column performance, reducing variability in peak shapes and retention times. This consistency minimizes technical artifacts, enabling clearer biological insights when comparing samples.

Various methods are commonly used to determine protein concentration. The Bradford Assay, which utilizes Coomassie Brilliant Blue dye, measures protein concentration through a colorimetric change, requiring a standard curve prepared with known protein concentrations to ensure precision. The bicinchoninic acid (BCA) assay forms a purple‐colored complex for protein quantification, with sensitivity optimized by adjusting reagent ratios and incubation conditions. Fluorescence‐based assays, such as the Qubit Protein Assay, use dye‐binding technology for highly sensitive quantification with minimal interference, making them suitable for samples with low protein concentrations.

The 2D Quant Kit is another option, which quantitatively precipitates proteins while leaving interfering substances in solution. This method produces a color density inversely related to protein concentration, with a linear response in the range of 0–50 µg and a volume range of 1–50 µL. When selecting a protein concentration method, it is important to consider the required sensitivity, dynamic range, and compatibility with buffer components, as some assays show varying tolerance to substances like SDS or protease inhibitors, including phenylmethylsulfonyl fluoride (PMSF).

If no suitable quantification assay is available, running sodium dodecyl‐sulfate polyacrylamide gel electrophoresis (SDS‐PAGE) can provide a rough estimate of protein abundance. Though less precise, this method offers a practical alternative for assessing protein concentrations in specific scenarios. This systematic approach ensures consistency and reliability in downstream analyses, especially when dealing with complex microbial samples containing proteins of varying abundances.

#### Protein digestion

Bottom‐up (shotgun) metaproteomic studies involve the enzymatic digestion of proteins into peptides, a process known as proteolysis, for untargeted protein identification. This method requires several preparatory steps to ensure efficient proteolysis. Initially, proteins are denatured using agents such as urea or guanidine hydrochloride to expose cleavage sites. Disulfide bonds are then reduced using reducing agents like dithiothreitol (DTT) or tris(2‐carboxyethyl)phosphine (TCEP). To prevent the re‐formation of disulfide bonds, cysteine residues are alkylated with agents like iodoacetamide, which react with sulfhydryl groups to form stable thioether adducts [127]. This alkylation introduces mass changes that must be accounted for during peptide identification, as discussed in Section Peptide identification with proteomics search engines.

Following these preparatory steps, proteins are enzymatically cleaved into peptides suitable for downstream LC‐MS/MS analysis [128]. The most commonly used protease is trypsin due to its high specificity and efficiency. It cleaves proteins at the C‐terminal side of lysine and arginine residues, producing peptides ideal for shotgun MS analysis. Lys‐C, another commonly used protease, complements trypsin digestion by cleaving at the C‐terminal side of lysine residues, particularly in high urea concentrations (8 M), enhancing peptide coverage. Alternative proteases such as chymotrypsin, Glu‐C, and Asp‐N may also be used to increase peptide diversity or for specific applications. However, the combination of trypsin and Lys‐C is often the most practical and widely applied choice.

The enzyme‐to‐substrate ratio is another important factor, with typical ratios ranging from 1:50 to 1:100 (w/w). Digestion time is also critical and usually involves incubating the proteome mixture at an appropriate temperature (e.g., 37°C) for several hours to overnight, depending on sample complexity and enzyme properties. Digestion is quenched by acidification, commonly using formic acid or trifluoroacetic acid to achieve a pH of 2–3. In methods such as S‐trap or FASP, peptides may also be eluted without an acidification step.

Peptide lysates are subsequently desalted or purified to remove salts and contaminants. Solid‐phase extraction (SPE), C18 ZipTips (Millipore), or ultrafiltration are commonly used for this purpose. In some cases, the desalting step can be omitted if peptides are desalted on a trap column in the liquid chromatography (LC) system.

Direct in‐solution protein digestion methods have been developed to streamline the workflow, offering efficient and high‐throughput options. Notable examples include SP3 [129], FASP [130], S‐trap [131], and a commercial kit based on the in‐StageTip (iST) [132]. These methods are designed to ensure high protein recovery and compatibility with downstream MS analysis, even when working with low protein amounts.

### Separation and fractionation techniques

Separation and fractionation enable researchers to reduce sample complexity and enhance the depth and sensitivity of protein identification and quantification. These processes can be performed at multiple levels, including the peptide, protein, and cellular stages, depending on the specific goals of the analysis [133]. Techniques such as peptide fractionation are frequently used to enhance LC‐MS/MS performance, while enrichment approaches allow for the targeted analysis of PTMs. At the protein or cellular level, fractionation strategies can further refine sample complexity or enrich specific components of interest.

#### On‐line and off‐line peptide fractionation

Peptide separation workflows can generally be categorized into one‐dimensional (1D) and two‐dimensional (2D) or multi‐dimensional approaches. In 1D‐LC, which is widely used in metaproteomics, reverse‐phase (RP) nano‐high‐performance liquid chromatography (nanoHPLC, mostly just abbreviated as LC or HPLC) employs C18 columns to separate peptides based on their hydrophobicity and is coupled directly with mass spectrometry for peptide analysis. 2D‐LC, often based on multidimensional protein identification technology (MudPIT) [134], combines strong cation exchange (SCX) with RP‐HPLC. Peptides are first fractionated on the SCX column based on their charge using salt or pH gradients for elution, and then further separated based on hydrophobicity on an RP‐HPLC column [135]. The 2D‐LC strategy has been applied in metaproteomic analyses to improve identification depth, with online 2D LC‐MS setups used for shotgun proteomics in studies of human gut and environmental microbiomes [135].

Off‐line pre‐fractionation, although less commonly used in metaproteomics due to its labor‐intensive nature and the increased MS time required, offers potential for deeper peptide and protein identification [136]. High‐pH RP chromatography is one such method and is orthogonal to low‐pH RP‐LC‐MS gradients. This fractionation can be achieved using either stage‐tip methods or HPLC systems. Stage‐tip‐based fractionation is straightforward to implement and is supported by commercially available kits (e.g., Pierce High pH Reversed‐Phase Peptide Fractionation Kit). On the other hand, micro‐flow HPLC systems enable higher‐resolution fractionation through continuous collection of numerous fractions and stepwise concatenation.

While extensive fractionation can significantly enhance the depth of metaproteomic analysis, it also increases costs, sample requirements, and instrument time, making it less feasible for large cohort studies. The adoption of multiplexing techniques, such as tandem mass tags (TMT) [137], has mitigated these limitations by reducing MS time and the required sample quantity per condition. The combination of off‐line peptide fractionation and multiplexing presents a promising and accessible option for researchers, particularly beginners, aiming to conduct in‐depth metaproteomic analyses to investigate microbiome functionality.

#### Enrichment of peptides with posttranslational modifications

PTMs are critical regulators of protein activity and function, and their study is uniquely possible through metaproteomics. Unlike other omics approaches, metaproteomics provides the direct capability to identify and quantify PTMs in microbial proteins, offering unparalleled insights into microbiome functionality. While analyzing PTMs at the metaproteome level is particularly challenging, several studies have successfully performed metaPTMomics on environmental and human gut microbiomes [70, 138, 139, 140]. These studies identified various PTMs, including methylation, hydroxylation, acylations, citrullination, deamination, phosphorylation, and nitrosylation, among others, with abundances varying across different microbiome types. Understanding the diversity and distribution of PTMs is essential for uncovering microbiome functionality. Recent advancements in the field have been detailed in two comprehensive reviews [141, 142].

Microbiome PTMs can be analyzed using non‐enriched samples combined with tailored bioinformatics workflows [138, 139] or quantitatively profiled using enrichment techniques at the peptide or protein level [70, 140]. Depending on the type of PTM, specific enrichment strategies may be employed to facilitate detection during MS analysis.

Immuno‐affinity enrichment is widely used for protein acylations, such as lysine acetylation, propionylation, and succinylation, and has recently been applied to human gut microbiomes [140]. This technique uses antibodies bound to agarose or magnetic beads to selectively enrich acylated peptides, improving MS sensitivity and specificity. However, this approach can be limited by the availability of motif‐specific antibodies and the inability to capture the full spectrum of modified peptides.

Immobilized metal affinity chromatography (IMAC) is a commonly used strategy in proteomics to enrich phosphorylated peptides for phosphoproteomic studies. Ti‐IMAC and Fe‐IMAC are typical examples, offering robust enrichment before LC‐MS/MS analysis [143].

Hydrophilic interaction liquid chromatography (HILIC) is another effective technique, particularly for enriching glycopeptides. This method capitalizes on its high selectivity and specificity for hydrophilic glycan moieties [144]. These enrichment approaches have been extensively applied to mammalian cells, tissues, and single bacterial strains, and they show potential for broader applications in microbiome studies.

#### Protein, cell‐level, and functional fractionation techniques

The high complexity of microbiomes often necessitates cellular and protein‐level separations to complement peptide‐level fractionation, enhancing the depth and resolution of metaproteomic analysis. Although high‐speed, high‐resolution mass spectrometers have made peptide fractionation sufficient for many proteomics workflows, the added complexity of microbiomes can still benefit from upstream fractionation approaches.

Capillary zone electrophoresis (CZE), a technique used to separate charged particles, shows promise for separating intact proteins and even bacterial cells [133]. Another method for separating proteomes from different bacteria is differential lysis, which, despite its relatively low granularity, can distinguish between bacterial types based on cell wall structure [117]. In this approach, sequential lysis is achieved using buffers of increasing strength, such as those containing urea or varying concentrations of SDS. This method can separate the proteomes of Gram‐negative bacteria, which have thinner cell walls, from those of Gram‐positive bacteria with thicker, multilayered cell walls [117].

For host‐associated microbiomes, removing abundant host cells is often critical to improving microbial signal detection. Techniques such as differential centrifugation and density gradient centrifugation [145] are commonly used to enrich microbial cells. Following lysis, additional separation of cellular components can be achieved through methods like ultracentrifugation [146], further increasing protein identification coverage.

Functional fractionation techniques, such as activity‐based protein probing (ABPP), can be used to study enzymatic functions at the proteome level [147]. ABPP employs small‐molecule probes that covalently bind to active sites of proteins with specific functions or residues. These labeled proteins can then be captured or enriched for LC‐MS/MS analysis, enabling detailed profiling of protein functions and aiding in drug target discovery. ABPP is particularly useful for annotating proteins with unknown functions [148], making it a relevant approach in microbiome studies. Recent applications of ABPP in both host‐associated and environmental microbiomes have uncovered diverse microbial enzymes, including thiol‐containing proteases, bile salt hydrolases (BSHs), glycoside hydrolases (GHs), and β‐glucuronidases [149].

### Automation

High‐throughput techniques have transformed sample preparation, simplifying labor‐intensive steps and revolutionizing workflows in proteomics, especially as datasets continue to grow in scale and complexity [150, 151]. These advancements have facilitated applications such as chemical proteomics [152], biomarker detection [153], and drug target discovery [154]. Although automation in metaproteomics has not advanced as rapidly as in proteomics, its potential for transforming the field is immense.

Automating metaproteomics workflows offers multiple benefits, including reduced sample handling time, minimized operator‐induced variability, and enhanced reproducibility. These improvements provide broader coverage of microbiome responses to environmental factors within limited experimental timeframes. Furthermore, high‐throughput automated workflows allow researchers to scale up the discovery of microbiome‐associated biomarkers and explore dynamic functional landscapes across diverse microbiomes. Automation also generates large datasets, enabling the application of artificial intelligence (AI) to uncover hidden patterns within metaproteomic profiles.

Automated sample processing in metaproteomics can be broadly divided into four key steps: microbial cell disruption and protein extraction, protein digestion and peptide clean‐up, and multiplexing.

#### Microbial cell disruption and protein extraction

In certain scenarios, such as working with complex clinical samples like human stool or saliva, microbial cell enrichment is often required but poses significant challenges. Sample properties can vary greatly within a data set, complicating efforts to standardize technical parameters for automated microbial cell purification. As a result, current automated metaproteomics workflows often exclude fully automated raw sample handling steps. For example, the RapidAIM 2.0 pipeline [155] includes manual bacterial enrichment and cell washing, with a 96‐channel liquid handler accelerating pipetting steps. In contrast, the SHT‐Pro protocol [107], the first high‐throughput pipeline specifically designed for large‐scale stool sample processing, begins with the lysis of raw stool samples without prior microbial enrichment. This approach is particularly beneficial when both host and microbial proteins are of interest.

Microbial cell disruption for protein extraction can be effectively automated in a 96‐well format using ultra‐sonication devices designed for high‐throughput workflows. These instruments facilitate efficient protein extraction, enabling downstream high‐throughput protein clean‐up. Several methods, including FASP, SP3, and S‐Trap, have been successfully adapted to microplate‐based formats, with studies showing that the combination of FASP and SP3 with iST yields the most robust results for high‐throughput protein processing [125].

#### Protein digestion and peptide clean‐up

Similar to manual metaproteomics workflows, automated protein preparation typically involves protein denaturation, reduction, alkylation, and protease digestion. These steps are relatively straightforward to automate and can be performed using liquid handling platforms equipped with low‐volume pipetting accuracy and heater‐shaker capabilities. Therefore, protein digestion is often considered one of the least complex steps to automate metaproteomic workflows.

Peptide clean‐up, however, presents greater challenges. Typically, this step is carried out manually by skilled personnel using solid‐phase extraction (SPE), C18 ZipTips, or ultrafiltration, as described in Section Protein digestion. During automation, sample heterogeneity at this stage can introduce variability, complicating experimental parameter control. A promising solution involves replacing centrifugation through reverse‐phase columns with pipette‐based mixing of reverse‐phase resins. This approach has been incorporated into workflows like RapidAIM 2.0 [155] and is supported by established proteomics automation protocols. For example, the autoSISPROT system offers all‐in‐tip sample preparation capabilities, demonstrating compatibility with automated platforms [154].

#### Multiplexing

The integration of automated sample handling with techniques like tandem mass tag (TMT) labeling significantly enhances throughput and accelerates the discovery process in metaproteomics. However, the high cost of TMT reagents might be a challenge for broader applications. One solution involves pre‐aliquoting and drying TMT reagents in a 96‐well plate format, a strategy that reduces reagent waste and preparation time. This approach is compatible with automated workflows, such as those used in the RapidAIM 2.0 platform, and facilitates more efficient reagent utilization [155].

While advancements in automation have enabled notable progress in metaproteomics, most current systems are semi‐automated rather than fully automated. Continued development of automation technologies is essential to further streamline workflows, enhance sample processing speed, and achieve higher throughput.

### Mass spectrometry data acquisition methods

Mass spectrometry analysis of (meta)proteomes is predominantly carried out using (HP)LC‐MS/MS. A fundamental limitation of mass spectrometers, even when combined with multidimensional separations, is their inability to generate fragmentation spectra (or MS/MS spectra) for all peptides in a sample within a single run. This constraint has led to the widespread adoption of data‐dependent acquisition (DDA) as the dominant approach in proteomics over the past 25 years.

DDA, as discussed in Section DDA, involves selecting the most abundant precursor ions from the MS1 spectra for fragmentation in the MS2 (or MS/MS) stage, dynamically excluding previously fragmented ions to prioritize unfragmented targets. This strategy increases the diversity of identified peptides and proteins. In metaproteomics, however, the complexity of the samples presents significant challenges for DDA, particularly in achieving comprehensive sequencing depth and coverage. Even with the latest high‐resolution and highly sensitive mass spectrometers, DDA is inherently biased toward the most abundant ions, leaving many lower‐abundance peptides uncharacterized. Nevertheless, DDA remains the most widely used method due to its extensive validation, established workflows, and compatibility with a broad range of analytical tools.

Data‐independent acquisition (DIA), as discussed in Section DIA, is a more recent advancement that offers an alternative approach by fragmenting all peptide ions within predefined mass‐to‐charge (*m*/*z*) windows, rather than selectively targeting the most abundant ones. DIA addresses some of the limitations of DDA, particularly in terms of peptide coverage and reproducibility, making it increasingly attractive for metaproteomics. However, the broader data capture in DIA results in significantly more complex datasets that require advanced computational tools for processing and analysis. While progress has been made in developing such tools, further validation and optimization are needed before DIA can become a routine method for metaproteomics.

Both DDA and DIA have distinct advantages and limitations, and their choice depends on the specific goals of the experiment, the complexity of the sample, and the available computational resources.

#### DDA

DDA is the most widely used method in proteomics, particularly in shotgun proteomics, for identifying peptides in biological samples. In DDA mode, the mass spectrometer dynamically selects a specified number of the most abundant precursor ions (commonly referred to as the “topN”) for fragmentation. This prioritization ensures that the most intense ions within each acquisition cycle are fragmented into smaller ions, generating MS/MS spectra that serve as unique fingerprints for peptide identification. To enhance the detection of lower‐abundance peptides, DDA incorporates a process known as dynamic exclusion. Previously selected precursor ions are temporarily excluded from subsequent fragmentation, increasing the diversity of peptides analyzed within a single run. These MS/MS spectra are then analyzed using proteomics software packages (Section Peptide identification with proteomics search engines).

DDA has several advantages, making it a popular choice for metaproteomics workflows. It is relatively simple to configure and analyze compared to more complex approaches like DIA, making it accessible for both beginners and experienced researchers. The one‐to‐one relationship between spectra and peptides reduces computational demands during data analysis, particularly when a well‐curated protein database is available. More information on creating a protein database is provided in Section Database construction or selection. Furthermore, DDA supports relative quantification of proteins using both label‐free quantification (LFQ) and labeling approaches, offering flexibility for various experimental designs (Section Protein quantification). Its longstanding use in proteomics has also led to the development of numerous software tools and well‐established workflows, enhancing its reliability and versatility.

Despite its strengths, DDA has notable limitations. Its reliance on selecting the most intense precursor ions means that low‐abundance proteins may go undetected, especially in complex samples. Additionally, DDA often fails to identify the same peptides consistently across multiple runs, resulting in missing values for low‐abundance proteins and complicating large‐scale quantitative studies.

Overall, while DDA is not without its limitations, it remains the most widely used and versatile technique in metaproteomics [156]. For studies requiring deeper proteome coverage or greater reproducibility, alternative methods like DIA may offer complementary advantages.

#### DIA

DIA mass spectrometry has emerged as a powerful approach in proteomics, providing broad protein coverage, high reproducibility, and quantitative accuracy. Unlike DDA, which focuses on fragmenting a limited number of the most intense precursor ions, DIA fragments all ions within predefined *m*/*z* windows. These windows are repeatedly scanned across the entire *m*/*z* range, generating complex MS/MS spectra that provide a more comprehensive view of the proteome. This inclusivity is particularly advantageous in metaproteomics, where samples contain an overwhelming diversity of peptides and low‐abundance proteins that might be missed by DDA.

DIA has demonstrated significant potential in metaproteomics applications. Its application in metaproteomics was first evaluated in gut microbiome studies [157] and has since expanded to various contexts, including Chinese liquor fermenter starters [158], and multicenter diagnostic research on tongue coating samples for gastric cancer [63]. Recent advances in MS instrumentation, such as data‐independent acquisition—parallel accumulation serial fragmentation (DIA‐PASEF) [36] and the Orbitrap Astral [37], have significantly improved DIA's sensitivity and resolution, enabling deeper proteome coverage in highly complex microbial communities.

One of DIA's key advantages lies in its ability to capture a broader range of peptides compared to DDA, enabling deeper proteome coverage and improved detection of low‐abundance proteins [36, 63, 157, 158, 159, 160, 161]. Another significant advantage is its reproducibility across samples, as it is less susceptible to variations in ionization efficiency [162]. This consistency makes DIA particularly well‐suited for large‐scale quantitative studies.

Despite its advantages, DIA also comes with challenges, particularly in data analysis. Indeed, analyzing the complex MS/MS spectra generated by DIA requires advanced computational tools and specialized expertise which is further discussed in Section Peptide identification with proteomics search engines. Additionally, because DIA fragments all ions within a given *m*/*z* window simultaneously, the resulting spectra are more complex and less specific to individual peptides compared to DDA. This reduced specificity can make it challenging to confidently resolve detailed structural or sequence‐level information for single peptides, limiting DIA's utility for applications that require precise characterization, such as studying PTMs or differentiating highly similar peptide sequences. These inherent trade‐offs highlight the importance of carefully tailoring DIA workflows to specific research objectives.

Nevertheless, DIA's rapid advancements make it a promising tool for metaproteomics, providing the depth and reproducibility required to explore the functional landscape of microbial communities comprehensively.

#### Critical parameters to optimize the HPLC and MS methods

Optimization of HPLC and MS methods is crucial for obtaining high‐quality data in metaproteomics workflows. Each parameter below plays a significant role in ensuring accurate peptide separation, identification, and quantification. Metaproteomics, with its added complexity compared to standard proteomics workflows, requires specific adjustments to many of these parameters.

(i) Analytical column quality, gradient and flow rates

Peptides are commonly separated using HPLC, which is directly coupled to the MS, using either commercial or in‐house analytical HPLC columns. These separations are achieved with a mobile phase composed of increasing concentrations of acetonitrile (ACN). For laboratories using in‐house columns, stringent QC checks are crucial to ensure consistent column performance, as explained in Section Quality control of LC‐MS/MS.

Metaproteomics samples present significantly greater chromatographic challenges than single‐species proteomics due to their inherent complexity [163]. To address this, typical mobile phase gradients of 5%–35% of 80% ACN or 5%–30% of 100% ACN over 1–2 h are generally sufficient for tryptic peptide elution. However, adjustments may be required for specific experimental setups. For example, chemically labeled digests with increased hydrophobicity often require a steeper gradient with a higher final concentration of ACN for complete peptide elution.

Efficient gradient design is essential to optimize runtime and achieve an even distribution of peptide elution across the gradient. Since fewer peptides elute at the beginning and end of the gradient, tailoring the gradient can improve separation and detection [164]. Accurate peptide quantification requires sufficient sampling points per LC peak, making short gradients (e.g., 10‐min gradients) generally unsuitable for metaproteomics in DDA mode. Comprehensive tutorials on gradient optimization are available for general proteomics [165], and metaproteomics specifically [166].

LC flow rates typically range from 200 to 300 nL/min. Recently, higher flow rates have gained popularity to accelerate sample duty cycles. However, these higher flow rates compromise sensitivity. Strategies to offset this limitation include increasing the sample loading amount or using dimethyl sulfoxide to boost signal intensity, making higher flow rates more viable for metaproteomics workflows.

(ii) MS settings in DDA workflows

Optimizing MS parameters plays a key role in obtaining high‐quality data in metaproteomics. While those new to the field are generally not expected to configure MS settings, understanding key optimization steps can provide valuable context for interpreting data and troubleshooting issues.

Accurate mass measurements require regular calibration of the mass spectrometer, which is crucial for reliable peptide identification and quantification. Additionally, source parameters such as source temperature, flow rates, and nebulizer gas pressure must be optimized to enhance ionization efficiency and maximize signal intensity. The specific optimization steps vary depending on the type of mass analyzer used, such as time‐of‐flight (TOF) or Orbitrap instruments. Key parameters for these analyzers include scan range, resolution, and scan speed, which must be fine‐tuned to ensure precise mass measurements and resolve closely spaced peptide ions. Similarly, collision energy settings for peptide fragmentation need careful adjustment to generate high‐quality fragment spectra for peptide identification.

Dynamic exclusion is a critical parameter in DDA workflows, requiring careful calibration to align with the chromatographic gradient and peak width. This setting prevents repeated fragmentation of the same peptide by excluding it temporarily after its initial fragmentation, thereby increasing peptide diversity. However, this approach poses challenges, particularly in metaproteomics. Many researchers rely on spectral counting for relative quantification, as it has been shown robust for metaproteomic datasets with significant differences in cell numbers and total protein amounts between community members [167]. Nonetheless, dynamic exclusion can limit the number of spectra acquired for abundant peptides, leading to fewer spectral counts than expected and potentially skewing quantification accuracy. This issue is exacerbated with modern high‐resolution instruments, where the correlation between peptide abundance and peptide‐spectrum matches (PSMs) becomes less relevant due to faster scan rates and increased resolving power. Dynamic exclusion times must therefore strike a balance, ensuring high‐quality fragmentation spectra while maximizing the diversity of peptides analyzed. The choice between spectral counting and MS1‐based quantification methods like area under the curve (AUC) remains a topic of debate in metaproteomics.

In DDA, selecting the isolation window width for precursor ions is a critical optimization step. A wider isolation window, up to 2 Da, allows the collection of more ions, resulting in higher‐quality MS spectra. However, this increases the risk of generating chimeric spectra, where fragments from multiple precursor ions are combined, complicating peptide identification. Conversely, narrower isolation windows, down to 0.7 Da, reduce the likelihood of chimeric spectra but limit the number of ions isolated, potentially impacting signal intensity. In metaproteomics, the high density and diversity of precursor ions in certain mass ranges complicate this balance, as even narrow windows can capture multiple ions. Advances in mass spectrometers, such as faster scan speeds, now enable higher topN settings in DDA workflows, helping to address this challenge by acquiring more fragmentation spectra within a given run.

(iii) MS settings in DIA workflows

Optimizing DIA workflows requires careful calibration of several key parameters to achieve accurate and comprehensive peptide identification. The width of mass isolation windows is particularly critical, as narrower windows, such as 2 *m*/*z*, provide higher resolution and more precise fragmentation spectra, which are essential for resolving complex peptide mixtures. However, narrower windows can reduce proteome coverage, as fewer ions are isolated in each cycle. Balancing resolution with proteome coverage is thus a central challenge in DIA optimization. Recent advancements, such as the Orbitrap Astral mass spectrometer, support exceptionally narrow isolation windows while maintaining high scanning speeds, effectively bridging the gap between DDA and DIA methodologies.

In addition to tuning isolation windows, optimizing collision energy is required for generating high‐quality fragment ions, while chromatographic conditions, including gradient length and flow rate, must be carefully calibrated to align with the DIA cycle time. Ensuring sufficient acquisition points across peptide elution peaks is essential for accurate quantification and peptide identification. DIA workflows in metaproteomics are advancing rapidly, providing enhanced resolution and deeper proteome coverage in complex microbial samples [37, 168]. Detailed guidelines for these optimization strategies can be found in recent studies exploring advancements in DIA methodologies [169, 170, 171].

#### Quality control of LC‐MS/MS

A comprehensive QC workflow begins with a blank injection of solvent without any sample to check for background contamination. Ideally, a blank run should produce minimal identifications, which can be verified visually or through database searches. Contamination sources can include transport solvents used in HPLC systems, so these should be carefully monitored. Next, a standard injection of a known peptide mixture, such as cytochrome C or bovine serum albumin (BSA) digest, is performed to confirm instrument calibration and performance. Simple mixtures like these are useful for testing HPLC performance, while more complex peptide mixtures, such as HeLa digest, assess the mass spectrometer's ability to analyze complex samples. A representative microbiome sample digest can also be injected to refine the LC gradient profile, and such standards should be injected regularly throughout the run. Additionally, using reference microbiome material as a positive control can help verify the efficiency of protein extraction protocols. This ensures that the extraction method reliably captures a representative set of proteins from the sample, which is particularly important for metaproteomic studies. Database searches on complex standards should be used to monitor metrics like number of PSMs, peptide and protein identifications. Consistently tracking these values over time helps detect performance declines, signaling when the instrument requires cleaning or recalibration.

During the LC‐MS/MS run, retention times for known peaks should be monitored closely, as significant shifts compared to previous runs may indicate issues such as column blockage, connector leakage, or valve wear. Similarly, column back pressure should be monitored as a potential indicator of problems. Peak shape should also be evaluated for symmetry and sharpness; tailing or broadening peaks may suggest problems with chromatography or ionization efficiency. Signal intensity is another important parameter, and any significant drop compared to expected values may point to reduced instrument sensitivity or ionization issues.

After the run, each raw file must be carefully reviewed to identify potential issues. Failed runs should be rerun immediately to avoid batch effects caused by delayed reanalysis. The total ion current (TIC) chromatogram provides valuable information on instrument performance, and it should be examined for unexpected peaks or a noisy baseline, both of which may point to contamination or hardware issues. The base peak chromatogram provides additional insights into LC resolution. Comparing the TIC‐to‐base peak intensity ratio is also informative, as higher values often reflect increased sample complexity or poor chromatographic performance. Retention times and peak intensities across samples should be consistent, indicating good repeatability. Additional QC checks, such as principal component analysis (PCA) or heatmaps, can help pinpoint variations between runs and ensure data quality.

Metrics collected after protein identification and quantification are also essential for evaluating QC [172]. For example, the number of identified PSMs to the total number of MS2 spectra, the PSM identification rate, serves as a key indicator of data quality. Using a 1‐h gradient on a Q‐Exactive mass spectrometer with optimized conditions and high‐quality sample preparation, metaproteomic samples can achieve an ID rate of approximately 50%, meaning that 50% of spectra yield identified peptide sequences after 1% FDR filtering. Note that for samples in less trivial environments, such as soil, the PSM identification rate will be much lower. It is crucial to analyze high‐quality QC samples using the same LC‐MS/MS methods, as the identification rate depends heavily on both the instrument's performance and sample preparation.

In large‐scale projects lasting several weeks, retention time drift and signal drops are common. Blocking and randomizing samples during analysis is recommended to reduce systematic biases caused by these performance variations [173]. Implementing rigorous QC procedures at each step of LC‐MS/MS is essential to maintain data reliability and consistency, with standardized QC samples serving as valuable benchmarks for long‐term performance evaluation.

Several dedicated QC tools, such as MaCProQC [174], QCloud2 [175], and Rawtools [176], are available to evaluate the quality of LC‐MS/MS data. These tools provide a range of functionalities, from tracking performance metrics to generating clustering analyses for data quality evaluation. However, more recently, the HUPO‐PSI Quality Control working group has introduced the mzQC file format, a JSON‐based standard designed to streamline the reporting and exchange of MS quality control metrics. To facilitate adoption, they have also developed open‐source software libraries in Python (pymzqc), R (rmzqc), and Java (jmzqc), which provide functionalities for creating, validating, and analyzing mzQC files. These libraries enable researchers to integrate mzQC into diverse workflows for proteomics, metabolomics, and other MS applications, ensuring consistent data quality assessment and fostering interoperability across different analytical platforms [177].

#### Data management and data sharing

Effective data management and sharing are essential to advancing metaproteomics research, ensuring data integrity, reproducibility, and collaboration. A robust data management plan should include secure, redundant storage solutions to protect against data loss, particularly for large‐scale studies conducted over extended periods. Implementing version control for raw and processed data facilitates systematic tracking of updates and reanalyses, improving reproducibility and transparency.

Adhering to community standards, such as those established by the Human Proteome Organization Proteomics Standards Initiative (HUPO‐PSI) [178], is crucial for consistency and interoperability. The HUPO‐PSI defines data representation standards in proteomics to facilitate data comparison, exchange, and verification. Using standardized formats like mzML for mass spectrometry data [179], mzIdentML for identification results [180], and the Universal Spectrum Identifier (USI) for referring to any mass spectrum in publicly deposited proteomics datasets [181], ensures compatibility across platforms and tools, thereby streamlining collaborative efforts and enabling more efficient data use.

Metadata plays a critical role in making datasets interpretable, reusable, and comparable across studies. Comprehensive metadata should capture sample origins, preparation protocols, instrument settings, and data processing workflows, ideally using standardized ontologies like PSI‐MS Ontology. In proteomics, this information is collected in the Sample and Data Relationship Format for Proteomics (SDRF‐Proteomics) format, which provides a structured, tab‐delimited format for describing the relationships between samples and data files, mirroring the experimental workflow in proteomics [182]. Tools like lesSDRF offer user‐friendly interfaces to annotate metadata in SDRF format, facilitating standardization [183]. Recognizing the added complexity of microbial environments, the Metaproteomics Initiative is developing SDRF‐Proteomics templates tailored for metaproteomics, as current formats for single‐species proteomics do not fully address the nuances of microbial data. Standardized metadata not only supports computational analyses but also ensures structured inputs for machine learning models, advancing reproducibility and consistency across the field.

Depositing both data and metadata in recognized international ProteomeXchange repositories [184], such as PRIDE [185], aligns with the FAIR (Findable, Accessible, Interoperable, and Reusable) principles, promoting open science and innovation. These repositories make data accessible to the broader research community, enabling others to validate findings, conduct systematic reviews, and perform large‐scale analyses. Sharing practices in metaproteomics helps with benchmarking studies, development of new interpretation tools, and the ability to draw broader conclusions, significantly improving the field's collaborative potential and impact.

## COMPUTATIONAL ANALYSIS OF METAPROTEOMICS DATA

### Peptide identification, protein inference, and quantification

After acquiring MS/MS spectra from mass spectrometry, the next step is to identify the peptides present in the sample. This involves analyzing the fragmentation patterns in the MS/MS spectra to determine the specific amino acid sequences of the peptides. This process is performed using search engines, often integrated into comprehensive proteomics software packages (Section Peptide identification with proteomics search engines). Typically, these algorithms match the experimental MS/MS spectra to a theoretical protein sequence database, and the success of this step depends heavily on the selection or construction of an appropriate database, as outlined in Section Database construction or selection. The search engine then applies a false discovery rate (FDR) threshold to filter out postential false positives (Section PSM FDR control). Peptides passing this filter are subsequently used for protein inference (Section Protein inference) and quantification (Section Protein quantification). All these sections focus on DDA MS, while Section DIA data analysis is dedicated to tools specifically designed for analyzing DIA MS data.

#### Peptide identification with proteomics search engines

Shotgun metaproteomics experiments generate large datasets of MS1 and MS2 spectra, which form the basis for downstream analysis. With advancements in high‐throughput MS, these datasets now range from thousands to millions of spectra, making manual interpretation impractical. To address this challenge, search engines are essential for interpreting the data and identifying peptides. Peptide identification relies on three main strategies: (i) sequence database searching, where experimental spectra are matched to theoretical spectra derived from protein or peptide sequences in a database; (ii) de novo sequencing, which directly infers peptide sequences from spectra without a reference database; and (iii) spectral library searching, where experimental spectra are compared to curated libraries of previously validated spectra. These methods are often complemented by post‐processing steps to enhance accuracy and confidence in peptide identification, as outlined in Section PSM FDR control. Additionally, most proteomics software packages integrate peptide identification with protein inference and quantification, a topic discussed in Sections Protein inference and Protein quantification. Some specific metaproteomics software also integrates taxonomic and functional analyses, as outlined in Section Taxonomic and functional analysis.

(i) Protein sequence database searching

Database search algorithms are fundamental for interpreting mass spectrometry data, particularly in metaproteomics, where the complexity of microbial communities poses significant analytical challenges. These algorithms match experimental MS/MS spectra to theoretical spectra generated from protein sequence databases. The success of this process depends on the choice of search engine, the search parameters used, and the composition of the database, all of which influence the number and type of peptides and proteins detected.

Database search engines start by using a selected reference protein sequence database, which is in silico digested to emulate the cleavage rules of the enzyme used during protein digestion, most commonly trypsin. From these digested sequences, theoretical MS/MS spectra are generated and compared to the experimental MS/MS spectra obtained during mass spectrometry. Each combination of theoretical peptide and spectrum (peptide‐spectrum match, PSM) is assigned a similarity score, with the search engine ranking and filtering potential PSMs based on the score and peptide properties. The exact method of score calculation varies between search engines, and these differences can affect both sensitivity and specificity. An in‐depth explanation of the various scoring algorithms used in database search engines can be found in this comprehensive review [186].

Each database search engine offers unique advantages and limitations, including variations in processing speed, compatibility with input and output formats, support for post‐processing tools, and overall user‐friendliness. These factors significantly influence their performance in metaproteomics workflows, where the complexity and scale of datasets demand highly efficient and reliable analysis tools. A detailed discussion of these tools and their applications is available in a comprehensive review [187]. A selection of database search engines and proteomics software commonly used in metaproteomics research is highlighted below:

SearchGUI [188] provides simultaneous access to multiple complementary search algorithms, including X!Tandem [189], Comet [190], Andromeda [191], OMSSA [192], Sage [193], and others. Its companion tool, PeptideShaker [194], seamlessly imports SearchGUI output and offers a comprehensive, user‐friendly interface for interpreting and visualizing results. Additionally, PeptideShaker includes a direct export feature to Unipept, enabling streamlined downstream taxonomic and functional analysis [195, 196]. A detailed tutorial is available on the CompOmics web page to guide users through these workflows [197]. Andromeda [191], used in MaxQuant [198], is widely used for its ease of use and MS1 quantitative capabilities. Users benefit from a well‐established community, including annual user meetings and a dedicated forum for support. Mascot (Matrix Science) and Proteome Discoverer (Thermo Fisher Scientific) are popular commercial tools with extensive user bases. FragPipe, using MSFragger [199], and pFind [200] incorporate open search strategies, which improve sensitivity by enabling the identification of PTMs. Sipros [201], ProteoStorm [202] and COMPIL 2.0 [203] are tailored specifically for metaproteomics but are perceived less user‐friendly than mainstream software. Tools such as Sage [193] and MSFragger [199] leverage advanced spectral and sequence indexing strategies to significantly accelerate database searches, making them highly promising for improving the speed of metaproteomics analysis.

For researchers who want more integrated solutions, several software suites can simplify metaproteomics workflows by consolidating multiple steps and managing the high density of information inherent to the field. Galaxy for Proteomics (Galaxy‐P) is another versatile platform offering numerous tools and workflows tailored to metaproteomics, including database generation, discovery analysis, verification, quantitation, and statistical analysis [204, 205, 206]. With public gateway availability [207] and access to training resources via the Galaxy Training Network [208], Galaxy‐P is a valuable resource for researchers seeking an open and user‐friendly platform for users to access metaproteomic workflows. The MetaProteomeAnalyzer (MPA) software suite [209] offers modules for protein database creation, database searching, protein grouping, annotation, and results visualization. Its user‐oriented design makes it a suitable option for both beginners and experienced researchers. MetaLab [210] is an integrated data processing pipeline that includes tools for sample‐specific database generation, peptide determination, taxonomic and functional profiling, and abundance analysis. Its open search strategy enables comprehensive profiling of PTMs and improved sensitivity. Additionally, MetaLab offers workflows for taxonomic analysis based on metagenome‐assembled genome (MAG) databases, allowing peptide‐to‐genome linkages for improved specificity compared to traditional lowest common ancestor (LCA) methods.

In these tools, selecting appropriate search parameters is essential for reliable and meaningful results. The choices regarding modifications, enzyme specificity, and mass tolerance significantly impact the identification of PSMs. Below are key considerations:Selection of modifications: It is important to distinguish between modifications introduced by the experimental workflow and biological modifications. Fixed modifications, like carbamidomethylation of cysteine, are commonly applied across all peptides to account for standard sample preparation artifacts, as discussed in Section Protein digestion. Variable modifications, such as methionine oxidation, are applied selectively to explore biologically relevant modifications. However, including too many variable modifications can expand the search space excessively, reducing identification rates. It is often best to limit variable modifications to the most biologically relevant ones.

Enzyme specificity and number of missed cleavages: Choosing the correct enzyme and setting an appropriate number of allowed missed cleavages affects the range of detectable peptides. For instance, trypsin, the most commonly used enzyme in proteomics, may occasionally miss cleavages after lysine (K) or arginine (R). Allowing one or two missed cleavages is generally a good compromise in metaproteomics, as it accounts for incomplete digestion without excessively broadening the search. Semi‐specific or nonspecific cleavage settings might be useful in some cases but can lead to longer processing times and a lower identification rate due to the expanded search space.

Mass tolerance: Mass tolerance settings should match the resolution capabilities of the mass spectrometer. For example, on a high‐resolution Q Exactive instrument with higher‐energy collisional dissociation (HCD) data, setting a precursor mass tolerance of 10 ppm (for MS1) and a fragment mass tolerance of 0.02 Da (for MS2) can balance accuracy and computational efficiency, restricting the search to relevant matches while taking advantage of the instrument's resolution.

Thoughtful parameter selection helps balance sensitivity and specificity, leading to high‐quality data that accurately reflects the sample's biological characteristics. Parameter adjustments should consider the mass spectrometer type, sample complexity, and specific research objectives.

(ii) De novo searching

De novo peptide sequencing assigns amino acid sequences to MS/MS spectra without requiring a protein sequence database for spectral matching. This approach provides an unbiased method for detecting peptides, independent of the quality and completeness of the protein sequence database. Several de novo sequencing algorithms have been introduced in recent years, including PEAKS, Casanovo [211], PepNovo [212], and the newly developed π‐HelixNovo [213], metaSpectraST [214], and NovoBridge [215].

When applied effectively, de novo sequencing can sensitively and accurately estimate the taxonomic composition and functional content of the microbiome without prior knowledge of the system under study. It also has the potential to identify unsequenced members of the microbial community. Furthermore, de novo sequencing can be used to evaluate the completeness and suitability of a protein sequence database for metaproteomics research [216]. Recently, the progress and opportunities in de novo sequencing for metaproteomics were reviewed, emphasizing its potential for unsequenced species detection and deeper functional insights into microbial communities [217].

Despite its promise, there remains a need for systematic benchmarking of de novo sequencing tools to assess their applicability to metaproteomics. In particular, most tools and approaches for de novo metaproteomic analysis still require some input from databases either to help selecting peptides or to gain information from the identified peptides. Evaluating their performance in terms of sensitivity, accuracy, and throughput is essential to ensure their effectiveness in the complex and diverse datasets characteristic of microbiome studies.

(iii) Spectral library searching

Spectral library search engines operate on principles similar to database searching but differ by directly comparing experimental MS/MS spectra to pre‐existing libraries of validated spectra. These libraries consist of MS/MS spectra previously acquired through the analysis of complex peptide mixtures and conventional sequence database searches or generated using predictive deep‐learning algorithms. Unlike sequence database searching, spectral library searching can incorporate additional parameters, such as retention time on the LC column and the relative intensities of fragment peaks within the spectra, enhancing both accuracy and confidence in peptide identification.

The development of AI‐based tools like MS²PIP [218] and Prosit [219] has made it possible to generate high‐quality spectral libraries from protein sequence databases [220]. These advancements have expanded the applicability of spectral library searches by enabling the generation of predictive libraries tailored to specific experiments. Newer spectral library search tools designed for DDA data, such as Mistle [221] and Scribe [222], have also emerged for metaproteomics research.

Spectral library searching offers a fast and efficient approach to match peptide sequences to MS/MS data, often outperforming traditional database searching in terms of speed and precision for well‐curated libraries. However, despite its potential, spectral library tools for metaproteomics require further evaluation, particularly regarding their usability and effectiveness for highly complex microbial data sets.

#### Database construction or selection

For single‐organism proteomics, constructing a protein sequence database is relatively straightforward, as it can be derived directly from the organism's genome. In metaproteomics, however, the complexity of microbial communities, the diversity of organisms, and the prevalence of unknown proteins present significant challenges. Selecting or generating an appropriate database is crucial, as the database must balance comprehensiveness and specificity. An incomplete database risks missing or falsely identifying proteins, while an excessively large database decreases the sensitivity of the analysis and inflates the FDR, as detailed in Section PSM FDR control [223, 224].

An optimal database for metaproteomics should be both comprehensive and specific. Comprehensive, as it should include all proteins potentially present in the sample. Missing sequences lead to false negatives, reducing peptide and protein identification rates. Specific, because it should exclude sequences unexpected to be present in the sample. Including irrelevant sequences increases random matches, inflates the FDR, and therefore negatively affects peptide (and protein) identification (see also Section PSM FDR control). Additionally, metaproteomic analyses often include contaminants from sample processing, such as leftover trypsin, BSA carry‐over, or keratin from handling. Incorporating these contaminants into the database, using resources like the common Repository of Adventitious Proteins (cRAP, https://www.thegpm.org/crap/), allows for their accurate identification and prevents misidentification with other proteins in the sample.

To create a suitable database, prior knowledge of the community composition is essential. This information can be derived from various sources, including prior literature, 16S rRNA amplicon sequencing, or metagenomic and/or metatranscriptomic sequencing, each offering different levels of resolution and success. Literature reviews provide only limited insights, whereas meta‐omics approaches offer the most comprehensive and detailed characterization of the community [225, 226, 227]. Additionally, depending on the sample's environment, host or dietary proteins may need to be included in the database. While adding these proteins can improve identification rates, it also increases database size and complexity, potentially complicating the analysis. The inclusion of nearly identical sequences, often inevitable in large databases, can further exacerbate protein inference issues (see Section Protein quantification). Sequence clustering algorithms [228] or protein grouping tools [229, 230] can address these challenges by consolidating redundant entries while retaining essential taxonomic and functional annotations.

The choice of database type depends on the sample type, the level of understanding of the microbial community, and the available resources. Based on these factors, different types of databases can be used, each with its own set of advantages and limitations (see Table 2). These include public repositories, reference catalogs, and meta‐omics databases, as detailed below.

**Table 2:** Comparison of database types for metaproteomics: public repositories, reference catalogs, and meta‐omics databases. The color indicates our preference: green represents favorable choices, yellow indicates intermediate choices, and red highlights unfavorable choices.

	Public repositories^a^	Reference catalogs	Meta‐omics databases
Monetary cost	Free	Free	Sample type dependent $100–$2000/sample or pooled samples
Time cost (labor & computation)	Days	Days	Genome‐resolved month–year, otherwise weeks
Comprehensiveness	Low to Medium depending on the sample representation in the repository	Medium to High depending on sequencing effort and multi‐omics integration	Medium to High depending on sequencing effort and multi‐omics integration
Identification probability	Low	Medium	High
Specificity	Low due to high diversity of the repository	Medium due to lack of strains resolution	High due to sample specificity
Misidentification probability	High	Medium	Low
Sequence Redundancy and Impact	High and difficult to resolve due to high diversity of the repository	Medium but can be resolved depending the curation level	Low and can be resolved as part of the metagenomic processing
Taxonomic Annotation and Resolution	Taxonomy not curated and potentially outdated	Depends on curation level (potential for misidentification due to closely related taxa)	Possibility of de novo annotation and species resolution based on metagenomic processing
Certainty/Applicability	Easily available but lacks the guarantee of appropriate sequences	Available for few sample types only and lacks of accuracy	High accuracy but requires particular expertise and extra time/cost

^a^ Restricted repositories have similar characteristics to reference catalogs in terms of specificity and sequence redundancy.

(i) Public repositories

Public repositories like UniProtKB [231] and NCBI RefSeq [232] provide extensive reference collections of protein sequences. However, these untailored (or unrestricted) databases often lack specificity and contain many unrelated sequences, leading to reduced identification rates and increased FDR (Section PSM FDR control). Furthermore, public repositories are biased toward well‐characterized microbes, such as model organisms or pathogens, and heavily studied environments or systems, such as clinical and human samples. This bias results in significant gaps for less‐studied environmental microbial communities, making these repositories incomplete for many metaproteomics applications. Filtering (or restricting) these repositories based on 16S rRNA analysis results can improve specificity, but the resolution of 16S rRNA sequencing is limited. Entire genera or sets of species often need to be included, preventing strain‐level specificity [233, 234].

(ii) Reference catalogs

Reference catalogs are curated collections of protein sequences tailored to specific environments or systems. They are available for well‐studied ecosystems such as the human gut [235, 236], the cow rumen [237, 238], and the mouse gut [239, 240, 241]. These catalogs are typically constructed by combining data from isolated microbes and metagenomic studies [242]. Although smaller and more targeted than public repositories, reference catalogs can still be relatively large for metaproteomic analyses and often aggregate data from many samples, including different individuals and studies—yet, not from the study itself, therefore also called unmatched meta‐omics databases. This composite nature introduces challenges, as even samples from similar environments can exhibit substantial variation in species composition and strain diversity. Consequently, reference catalogs can suffer from inaccuracies, incompleteness, and overrepresentation of certain subsamples [156, 243]. Like repositories, the specificity of reference catalogs can be improved by incorporating prior knowledge of the microbial community, such as results from 16S rRNA analysis, to narrow down the included sequences to those most relevant to the sample.

Alternatively, to address the challenges posed by large and composite catalogs, database‐reduction methods have been developed. These methods include the two‐step search approach [244], iterative workflows such as MetaPro‐IQ [120] and MetaLab [210], next to others. While these methods are often used in the field and increase the number of identified PSMs and peptides, some have been shown to significantly raise the number of false positives at both levels, exceeding the FDR estimate [245]. These methods should therefore be treated with caution, and additional validation might be appropriate before drawing biological conclusions.

(iii) (Matched) meta‐omics databases

Meta‐omics databases are constructed using metagenomic and/or metatranscriptomic data collected from the same sample as the metaproteomic analysis, making them the most specific databases available. These databases accurately reflect the species composition and strain diversity of the sample [11, 224, 246]. However, generating a high‐quality meta‐omics database requires significant sequencing effort, cost, computational resources, and technical expertise. Although the specific details of this process are beyond the scope of this manuscript, they have been extensively covered elsewhere [224, 247]. Briefly, constructing a meta‐omics database involves four key steps: sequencing, assembly, binning, and annotation.

To create a comprehensive database suitable for metaproteomic analysis, the sequencing effort must be sufficiently deep to capture the complexity of the community. One major advantage of meta‐omics databases is their ability to provide precise insights into the species and strain diversity of the sample, enabling direct linkage between genomes and identified proteins. This requires genome reconstruction through binning, where contigs are grouped into MAGs based on shared features. However, due to the complexity of microbial communities and limitations in sequencing depth, some MAGs may remain incomplete. Therefore, a robust meta‐omics database should include both binned and unbinned sequences to retain as much information as possible [248, 249].

Once reconstructed, MAGs and contigs are taxonomically annotated, and protein sequences or open‐reading frames (ORFs) are predicted and functionally annotated. The choice of tools and resources for these steps depends on the study's objectives [250]. Despite their specificity, meta‐omics databases can still be incomplete due to insufficient sequencing depth or the inability to recover all relevant MAGs from the sample. This issue can be partially addressed by performing exploratory 16S rRNA gene sequencing to assess the required sequencing depth for optimal metagenomic analysis [226].

Combining metagenomic data with metatranscriptomic data further improves the quality and specificity of the database [249, 251]. Since metatranscriptomics focuses on mRNA, it captures the active portion of the community, providing a gene‐centric view that aligns closely with the functional content of interest for metaproteomics.

#### PSM FDR control

A critical step in the process of peptide identification is acquiring a set of reliable PSMs. After PSMs are acquired, they are evaluated based on the scoring function of the search engine, retaining the highest‐ranked PSM for each spectrum—that is, the peptide sequence whose theoretical spectrum most closely matches the experimental MS/MS spectrum. However, regardless of the scoring algorithm used, some PSMs will inevitably represent false matches, making robust control of false positives essential.

**Figure 4:**
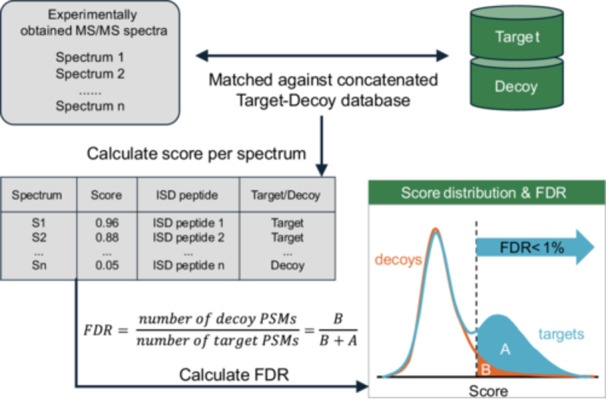
Principle of target‐decoy analysis and false discovery rate (FDR) calculation. (Top) The experimentally obtained MS/MS spectra are matched to in silico generated spectra of the concatenated target/decoy protein sequence database. (Middle) For each obtained spectrum, the match with the highest score is retained, together with the assigned in silico digested (ISD) peptide sequence and its target or decoy label. (Bottom) The score distribution is used to select which peptide‐spectrum matches (PSMs) will be considered as true matches. The metric to control the false positives is the FDR, and is calculated as the number of decoy PSMs divided by the number of target PSMs (in the Figure depicted as area B divided by the sum of areas B and A). Figure of (schematic) target/decoy distribution adjusted from Käll et al. [253].

The most commonly used strategy to manage false positives in (meta)proteomics is the target‐decoy approach [252]. In this approach, the protein sequences in the target database are processed in silico to emulate enzymatic digestion, generating theoretical peptides. The same procedure is applied to the reversed or shuffled sequences of a decoy database, ensuring that these decoy peptides are biologically implausible and not present in the sample. During the search, the experimental spectra are matched to both the target and decoy sequences in a concatenated target‐decoy database. This process results in PSMs being labeled as either target or decoy. The proportion of decoy PSMs in the final result serves as an estimate of the FDR, calculated as the number of decoy PSMs divided by the total number of accepted PSMs (Figure 4). The FDR is typically controlled at 1% in proteomics and metaproteomics experiments, but for highly complex samples such as soil microbiomes, the FDR threshold can be increased to 5% to retain a sufficient number of identifications for biological interpretation.

The specific challenges of metaproteomics add complexity to FDR control. The larger, more diverse protein sequence databases required for metaproteomics often increase the search space significantly, leading to a greater overlap between the score distributions of target and decoy PSMs. This overlap reduces the resolution of FDR estimation and necessitates careful database construction to limit irrelevant sequences, as discussed in Section Database construction or selection. Overly large but unspecific databases inflate the FDR by increasing random matches to both target and decoy sequences, resulting in fewer confident peptide identifications [187, 254]. Conversely, overly restrictive databases risk excluding true target sequences, resulting in missed matches, false negatives, and reduced proteome coverage. Therefore, achieving an optimal balance between database specificity and comprehensiveness is crucial to minimize false positives from decoy matches while maximizing target identifications, ensuring effective FDR control.

Metaproteomics workflows often rely on advanced post‐processing tools to improve the accuracy and confidence of peptide identifications. MS²Rescore [255] refines PSM scores by leveraging Percolator's search engine‐dependent features [256] while incorporating additional features derived from MS²PIP [257] and DeepLC [258]. By integrating these predictive features with Percolator's semi‐supervised machine learning model, MS²Rescore improves the separation between target and decoy PSMs, resulting in more accurate FDR estimation. These refinements not only increase peptide identification rates but also improve the reliability of downstream taxonomic and functional analyses, making them particularly valuable for complex microbiome data sets [259].

In metaproteomics, where samples often contain thousands of species, the challenge of FDR control is even larger by the inherent complexity and diversity of the microbial communities under study. Careful database construction (Section Database construction or selection), combined with robust FDR control during the search and advanced post‐processing techniques, is critical to ensure reliable peptide and protein identifications, thereby enabling meaningful biological insights from metaproteomics data sets.

#### Protein inference

Protein inference is a fundamental challenge in shotgun proteomics where the goal is to determine the proteins present in a sample based on the peptides identified through tandem mass spectrometry [223]. This process is complicated by the fact that peptides can often be mapped to multiple proteins or protein isoforms present in the commonly large protein database. This is especially the case in complex samples such as microbial communities where multiple species may contribute homologous proteins, making it difficult to conclusively infer which proteins are actually present [260].

**Figure 5:**
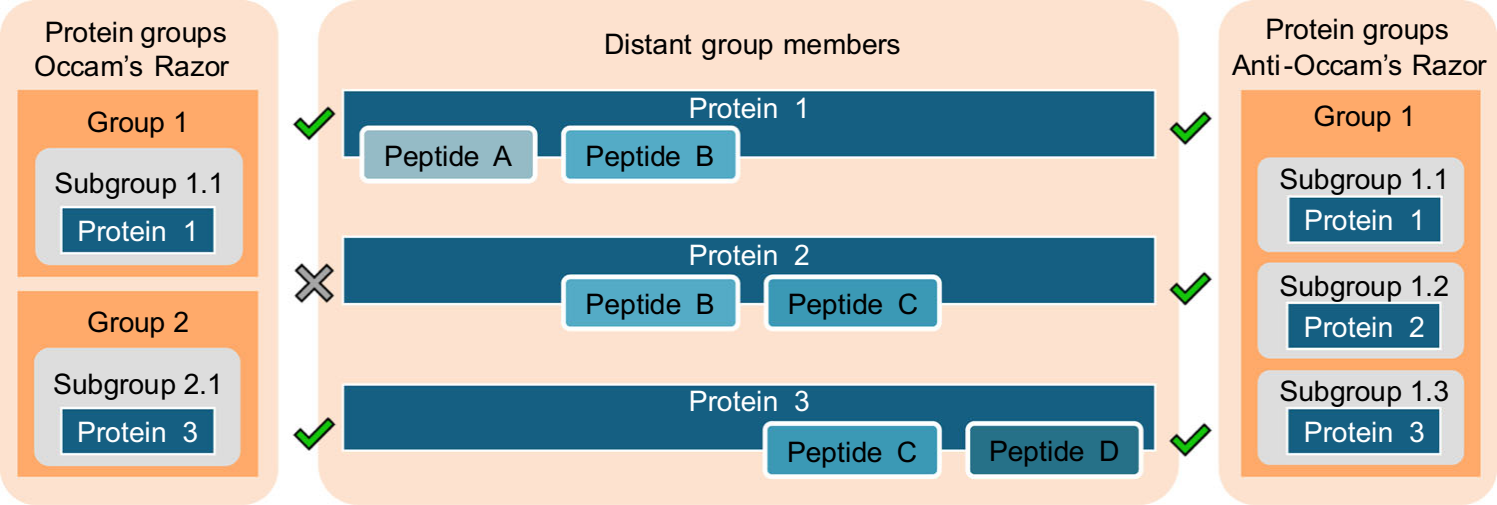
Practical example of (sub)grouping approaches. This grouping case deals with distant group members, meaning that certain proteins in the group don't share a single peptide, in this case proteins 1 and 3. Applying the rule of parsimony separates the group in this specific case. In the anti‐Occam case, protein 2 remains in a separate subgroup.

To address this complexity, protein grouping is commonly used to generate a more manageable list of identified protein (sub)groups for downstream analysis. However, different methods for protein grouping exist, as depicted in Figure 5, and these are typically performed by the search engine. It is essential to verify the default settings of the search engine to understand which grouping approach it applies, and if needed, adjust it to align with your research hypothesis. The two main approaches are Occam's razor and anti‐Occam's razor.

Occam's razor is based on the principle of maximum parsimony, providing the smallest set of proteins that can explain all observed peptides. However, this approach discards proteins not matched by a unique peptide, potentially losing their associated taxonomy and functions that might be present in the sample. Occam's razor is particularly suited for simpler, single‐species samples or targeted proteomics experiments, where reducing complexity is key.

In contrast, anti‐Occam's razor adopts a more inclusive strategy, retaining all proteins that can be mapped to at least one peptide, regardless of whether those peptides are shared with other proteins. This approach is beneficial for complex metaproteomic samples, where the goal is to capture as much protein diversity as possible. By being more inclusive, anti‐Occam's razor ensures that proteins from different species with minimal unique peptides are not overlooked, providing a more comprehensive picture of the microbial community. However, this inclusivity comes at the cost of increased complexity in the resulting protein list.

After choosing between Occam's and anti‐Occam's razor principles, proteins can then be grouped into protein groups or protein subgroups. Protein groups cluster proteins that share at least one peptide, offering a broader overview of potential protein identifications. Protein subgroups, on the other hand, are more specific and include proteins that share the exact same set of peptides. For example, the anti‐Occam's razor approach often benefits from subgrouping to prevent excessively large and uninformative protein groups. In metaproteomics, this approach helps disentangle the contributions of individual species, even when closely related proteins share substantial sequence similarity [260].

The choice of protein inference approach should align with the complexity of the sample and the research objectives. For single‐species or targeted studies, Occam's razor combined with protein grouping is advantageous for reducing false positives and simplifying downstream analyses. This strategy was used, for example, in analyzing the extended simplified human intestinal microbiota (SIHUMIx) mock community [261] as part of the CAMPI study [156]. For complex, multi‐species metaproteomic samples, anti‐Occam's razor combined with protein subgrouping is often preferred, as it maximizes protein diversity while maintaining manageable group sizes. This inclusive approach was used for fecal sample analysis in the CAMPI study [156]. Ultimately, the selection of a protein inference method depends on the specific characteristics of the sample and the research objectives. Researchers must balance the need for comprehensive protein identification with the practical considerations of data complexity and interpretability [260].

#### Protein quantification

Protein quantification is a central component of metaproteomics, offering valuable insights into the functional dynamics of microbial communities. By quantifying proteins, researchers can assess how microbes respond to environmental changes, revealing shifts in physiology and metabolic processes. For example, changes in nutrient availability can trigger significant alterations in protein expression within individual microbes [262] or entire microbial populations [263]. This section outlines the key concepts, strategies, and challenges in metaproteomic quantification, focusing on label‐free and labeling‐based approaches, as well as methods for downstream data analysis.

Metaproteomics workflows typically rely on two main quantification strategies: label‐free quantification (LFQ) and labeling‐based quantification. LFQ methods are widely used because they do not require stable isotope labels, making them more suitable for diverse and complex samples. Two common LFQ approaches are MS1 intensity‐based quantification and MS2 spectral counting. MS1 quantification measures precursor ion intensities by calculating the area under the curve or apex intensity for each identified peptide, with tools such as MaxQuant [264] or standalone alternatives like moFF [265] or FlashLFQ [266]. MS2 spectral counting, in contrast, quantifies peptides based on the number of matched MS2 spectra. Although simpler to implement, spectral counting typically has a narrower dynamic range and slightly lower precision. Currently, there is limited validation to determine which of the two primary quantification approaches—MS1 intensity‐based quantification or MS2 spectral counting—is more accurate for metaproteomics, or under which conditions one might outperform the other. One study demonstrated that spectral counting provided a more accurate measure of the proteinaceous biomass of members within a synthetic community compared to MS1 intensities [167]. Nonetheless, the prevailing consensus in the field suggests that both methods are generally suitable for metaproteomic quantification, with their applicability depending on the specific context and experimental goals.

Labeling‐based quantification approaches, while valuable in proteomics, are less commonly used in metaproteomics due to the complexity of microbial communities. These methods, including TMT and SILAC, enable absolute quantification and are particularly effective for controlled experimental designs requiring precise comparisons across samples. However, applying these methods to metaproteomics presents significant challenges. The diverse microbial populations and high sample complexity of environmental or clinical samples make labeling‐based approaches less practical, favoring label‐free strategies for most metaproteomics workflows. Nevertheless, labeling remains a viable option for targeted studies with well‐defined microbial communities.

Quantification in metaproteomics faces several challenges, particularly in aggregating peptide‐level data to infer protein abundances. This aggregation process is influenced by the protein inference problem [223], which determines how peptides are assigned to proteins or protein (sub)groups (see also Section Protein inference). Most software tools automatically assign peptides to proteins or protein groups, facilitating the quantification process. Once protein abundance data is obtained, normalization and transformation steps are crucial for meaningful statistical analysis. While various normalization methods have been proposed for proteomic data [267, 268, 269], the optimal approach for metaproteomics remains an area of active research.

One widely used normalization method, particularly for spectral count data, is the normalized spectral abundance factor (NSAF) [270]. This approach compensates for biases introduced by protein length and sample variability. It involves dividing a protein's PSM count by its amino acid length to account for protein size, followed by normalizing against the total PSM count within the sample to reduce between‐run batch effects. NSAF is relatively simple to calculate, robust to missing values, and particularly suited to the sparse data often encountered in metaproteomics. Further transformation, such as log or square root normalization, is typically applied to meet the assumptions of statistical tests.

A key distinction between standard proteomics and metaproteomics is the need to account for the diverse and complex nature of microbial communities. In metaproteomics, it may be advantageous to normalize protein abundances specifically for organisms or groups of organisms within the community. This targeted normalization allows researchers to focus on changes in gene expression and function within specific taxa, providing more granular insights into microbial activity. The normalized spectral abundance factor per organism (orgNSAF) normalization method has been proposed as a solution for this purpose, as it enables normalization of protein abundances within defined taxonomic groups [271, 272, 273].

A unique advantage of metaproteomic data is its ability to generate multiple data sets based on the research question. These data sets generally involve summing the abundance of constituent proteins into relevant categories. Broadly, there are three main categories: (i) individual proteins or groups of proteins with similar sequences, which can offer insights into the specific functionalities of individual organisms within the community; (ii) categories of biological functions assigned to proteins associated with the measured peptides, enabling researchers to investigate shifts in overall community functions; and (iii) taxonomic categories, where protein abundances can be used to estimate the relative contributions of different organisms within a microbial community.

The accuracy of both functional and taxonomic quantification is heavily dependent on the quality and completeness of protein annotations in the databases used. Functional categories can range from highly specific annotations, such as biochemical reactions, to broader descriptions of cellular processes like metabolism, gene expression, transport, or replication. Similarly, taxonomic quantification can achieve high resolution, down to the strain or species level [274, 275], but this depends on the depth and accuracy of protein annotations. In some cases, it is limited to higher taxonomic ranks when annotations are incomplete or ambiguous [43]. Metaproteomic measurements, when processed correctly, can provide an accurate representation of the relative proteinaceous biomass of microbial species within a community [167]. However, the specificity and accuracy of these measurements are closely tied to the reliability of the annotations used for protein classification [224, 254].

While these approaches enable the generation of robust data sets for understanding microbial abundance and function, further validation is necessary to refine these methodologies. Current quantification strategies in metaproteomics require additional benchmarking to identify optimal or equivalent approaches for various types of studies. Future research using mock communities with defined compositions and spike‐in proteins will be crucial for systematically evaluating the accuracy, reproducibility, and reliability of protein quantification methods in metaproteomics.

#### DIA data analysis

The application of DIA‐MS in metaproteomics, as discussed in Section DIA, demands tailored analytical workflows to manage the unique challenges posed by the complexity and scale of microbial communities. Unlike DDA, which prioritizes peptide selection, DIA generates complex spectra by fragmenting all ions within a predefined *m*/*z* range simultaneously. This comprehensive approach requires advanced computational tools and strategies to handle the resulting data.

Extracting quantitative and identification data from DIA‐MS involves specialized software, such as Spectronaut [276], DIA‐NN [277], and EncyclopeDIA [278]. These tools rely heavily on pre‐existing spectral libraries to match experimental spectra to theoretical peptides. Such libraries are often generated through prior DDA experiments or predicted from protein sequence databases. While promising, library‐free approaches that predict spectra directly from protein sequences remain computationally intensive and impractical for complex metaproteomics samples without additional data reduction strategies. One effective approach is using genome sequencing to limit the database search space or performing a preliminary DDA step to construct a targeted spectral library. These steps, although resource‐intensive, are essential for reducing ambiguity in protein and peptide identifications.

Metaproteomics data sets amplify the inherent analytical challenges of DIA‐MS due to their immense scale, which frequently involves millions of proteins and peptides. This complexity can lead to significant computational demands and requires extensive data processing pipelines. Direct library‐free DIA analysis for such data sets is virtually impossible with current technology unless supplemental genome sequencing or DDA‐based library construction is performed. These preparatory steps add complexity but are critical for optimizing DIA's utility in resolving the intricate dynamics of microbial communities.

Recent advancements in MS, including DIA‐PASEF [36] and the Orbitrap Astral analyzer [37], have shown potential for enhancing the application of DIA‐MS in metaproteomics. These technologies allow for deeper proteome coverage, improved sensitivity, and more accurate quantification. However, their integration into workflows must be carefully aligned with the computational tools and spectral library strategies mentioned above to fully exploit their capabilities.

A recent benchmarking study has demonstrated the reproducibility and accuracy of DIA‐MS for metaproteomic workflows in comparison to DDA‐MS methods [279]. Using mock communities of known taxonomic composition, DIA‐MS consistently identified and quantified more peptides and proteins across laboratories. Additionally, the reproducibility of protein and peptide identifications was higher in DIA‐MS workflows, which also provided accurate quantification of both protein abundances and taxonomic groups. These findings underscore the advantages of DIA‐MS for metaproteomics, including its capacity for deep sequencing, robust quantitation, and reproducibility across samples. However, current studies also highlight the limitations of existing DIA tools when applied to metaproteomic data sets, emphasizing the need for improvements in software capabilities to handle the unique complexities of microbiome samples. These insights stress the importance of optimizing library generation, computational tools, and workflows to fully leverage the potential of DIA‐MS for microbial community analysis.

Although DIA‐MS presents substantial benefits for reproducible and quantitative analysis, its application in metaproteomics is still evolving and faces several technical and computational challenges. Advances in mass spectrometry and bioinformatics hold promise for addressing these hurdles, enabling deeper insights into microbial community dynamics. Ongoing research is needed to refine workflows, optimize computational methods, and explore the potential of library‐free approaches to broaden their applicability in metaproteomics.

### Taxonomic and functional analysis

In metaproteomics, researchers aim to characterize microbial communities by determining the organisms present (taxonomic analysis) and elucidating their physiological roles (functional analysis). These analyses provide critical insights into the composition, diversity, and ecological functions of microbial communities across diverse environments. The accuracy of these assignments depends on the quality of peptide and protein identifications (see Section Peptide identification with proteomics search engines) and is significantly influenced by the choice of database (see Section Database construction or selection). Below, we describe the methodologies and tools available for taxonomic and functional annotation in metaproteomics, emphasizing the importance of robust annotation strategies and computational resources.

#### Taxonomic analysis

Taxonomic analysis in metaproteomics identifies the organisms present in a sample based on their expressed proteins. This analysis provides insights into microbial community composition and diversity, linking proteins to their taxonomic origins. Taxonomic assignment can be achieved using exact matching or homology‐based searches against comprehensive databases such as UniProtKB [231] or NCBI NR [232].

While numerous metaproteomics‐specific tools are available (described in Section Metaproteomics tools for taxonomic and functional analysis), researchers can also use tools originally developed for metagenomics, such as Centrifuge [280] and Kraken 2 [281]. These tools match peptides or proteins to known taxa, but their accuracy depends on the completeness of publicly available genome databases. If organisms in the sample have not been previously sequenced and deposited, taxonomic assignments may be incomplete or inaccurate.

Alternatively, taxonomic assignments can leverage meta‐omics databases derived from metagenomic assemblies. Proteins are inherently tied to genomes, and clustering metagenomic sequences into MAGs enables genome‐centric taxonomy assignment. Tools like GTDB‐Tk [282] use MAG taxonomy to assign taxa to proteins. For proteins not linked to MAGs, tools such as CAT [283] can infer taxonomy based on the context of all the genes in an assembled contig. Advances in long‐read sequencing are revolutionizing genome assembly from metagenomes, further improving taxonomic assignments [284].

#### Functional analysis

Functional analysis of metaproteomes reveals how microbial communities contribute to environmental processes, human health, and disease. By measuring the abundance of proteins involved in processes such as metabolism, transport, replication, and defense, functional analysis provides a window into microbial community dynamics and their roles in ecosystems.

To describe microbial functions, various functional ontologies are used: (i) Gene Ontology (GO): Organizes annotations into three categories: molecular functions, biological processes, and cellular components. GO terms are used to describe what a gene product does (molecular function), the biological goals it helps achieve (biological process), and where in the cell it acts (cellular component) [285]; (ii) Enzyme Commission (EC) numbers: Categorizes enzymes by the chemical reactions they catalyze, particularly useful in studies of enzymatic activity and the role these enzymes play in metabolic pathways; (iii) Kyoto Encyclopedia of Genes and Genomes (KEGG): Maps proteins to metabolic and signaling pathways, illustrating their interactions within larger biological systems [286].

There are also more specialized ontologies such as MEROPS [287] for proteases and CAZy [288] for carbohydrate‐active enzymes, including glycoside hydrolases, offer enhanced specificity for analyzing distinct functional categories within microbial communities.

Functional annotations can rely on computational tools commonly used in metagenomics, such as KoFamKOALA [289], InterProScan [290], and eggNOG‐mapper [291]. However, while these tools provide robust frameworks for mapping protein functions, more tailored tools specifically designed for the unique requirements of metaproteomics are available and discussed in Section Metaproteomics tools for taxonomic and functional analysis.

#### Peptide‐centric versus protein‐centric approach

In metaproteomics, taxonomic and functional analyses can be performed using either a peptide‐centric or protein‐centric approach. In the peptide‐centric approach, peptides identified through MS are directly annotated with taxa and functions based on their matches to in silico tryptic digests of known protein sequences. This approach ensures that all potential protein matches are retained during annotation, providing a broader view of possible taxa and functions. In the protein‐centric approach, peptides are first mapped to their corresponding proteins or protein (sub)groups, aggregating peptides that share common proteins. This step addresses the protein inference problem, a challenge in assigning peptides to proteins due to shared sequences among multiple proteins.

The peptide‐centric approach typically considers all proteins that a peptide could originate from, whereas protein‐centric tools may discard information deemed redundant based on the chosen protein (sub)grouping strategy. These different approaches may lead to variations in the resulting annotations, and the debate over which method provides the most accurate results remains an active topic in metaproteomics research [156].

#### Metaproteomics tools for taxonomic and functional analysis

**Figure 6:**
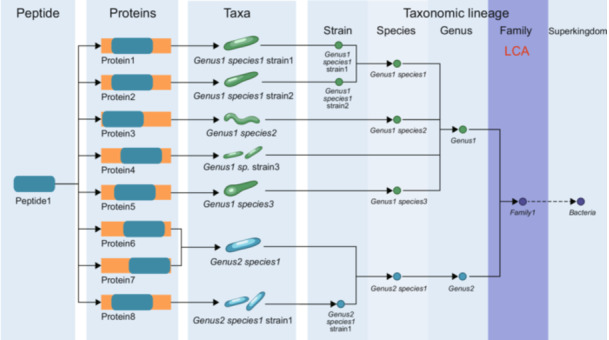
Calculation of the lowest common ancestor (LCA) for a tryptic peptide. In this figure, the hypothetical Peptide 1 is present in eight different proteins, which are associated with seven distinct organisms. The LCA for these organisms is identified as the hypothetical Family 1. Figure adjusted from Van Den Bosschee et al. [295].

Various tools have been developed for taxonomic and functional analysis in metaproteomics, each with distinct features and applications [292]. Unipept is a powerful ecosystem of tools for the taxonomic and functional analysis of metaproteomics samples, offering a command‐line interface (CLI), a desktop application, a web application, and an application programming interface (API) to accommodate diverse user preferences and workflows [195, 293, 294]. It follows a peptide‐centric approach, assigning taxa and functions directly to peptides by mapping them to the UniProtKB database. For taxonomic classification, Unipept calculates the LCA by identifying the most specific, or lowest, shared taxonomic rank among all taxa associated with a peptide's matched proteins (Figure 6). More details on how the LCA is calculated can be found in a recent comprehensive tutorial [295]. Unipept also supports extensive functional analysis by reporting functions based on the GO, EC, and InterPro classifications. For each peptide, it aggregates all annotations associated with proteins matching the input peptide and counts their occurrences. This information is displayed in a table within the web application. Detailed tutorials and examples for using Unipept have been published [295, 296], and the documentation available on the website (https://unipept.ugent.be/) offers additional guidance to help users navigate the tool.

The Peptonizer2000 is a novel metaproteomics pipeline for taxonomic inference that models the errors and uncertainties introduced by a typical metaproteomics analysis pipeline [297]. Indeed, the analysis of mass spectra is inherently challenging: researchers need to match observed data to databases of protein sequences, where factors such as database bias, ambiguous spectra, degenerate peptide sequences, and inter‐species sequence homology come into play. The Peptonizer2000 pipeline uses Bayesian statistics to model peptide sequences, associated taxa, and the possible errors and uncertainties introduced earlier as a graph. Subsequently, the Belief Propagation algorithm is utilized on this graph to compute probability scores that indicate the potential presence of a taxon in a sample under study.

MetaLab [210, 298, 299, 300] is an integrated software platform that provides a streamlined pipeline for microbial identification, quantification, and taxonomic profiling using mass spectrometry raw data. Employing a hybrid approach, MetaLab combines information derived from both peptide‐centric and protein‐centric metaproteomics analyses. MetaLab utilizes a precomputed index of the UniProtKB for taxonomic classification of identified peptides and retrieves functional annotations from the eggNOG database [301]. The latest version supports DDA and DIA workflows across various mass spectrometry platforms [302]. Comprehensive resources on iMetaLab [300] can be found on their dedicated Wiki‐page (https://wiki.imetalab.ca/).

Prophane [303] is a software tool designed for taxonomic and functional annotation of metaproteomes, offering interactive result visualization and an intuitive web‐based interface. It integrates data from various annotation databases, including NCBI [304], UniProtKB [231], eggNOG [301], or Pfam [305]. Unlike tools such as Unipept and MetaLab, Prophane adopts a purely protein‐centric approach for its analyses. The software is accessible both as a Conda package (https://anaconda.org/bioconda/prophane) and via a web service (https://prophane.de/login). Tutorials and example data sets are available on the tool's website (https://prophane.de/about/tutorial).

The MetaProteomeAnalyzer (MPA) [209] is an open‐source Java tool designed for the taxonomic and functional analysis of metaproteomics data. MPA employs both sequence‐based and spectral‐based approaches to identify organisms and functional pathways in a sample, enabling researchers to explore the metabolic activities of microbial communities and their environmental interactions. The software supports multiple search engines and incorporates features to reduce data redundancy by grouping protein hits into so‐called meta‐proteins. MPA is available as a desktop application, and extensive tutorials, documentation, and other resources are provided on its homepage (www.mpa.ovgu.de).

### Downstream statistics

**Figure 7:**
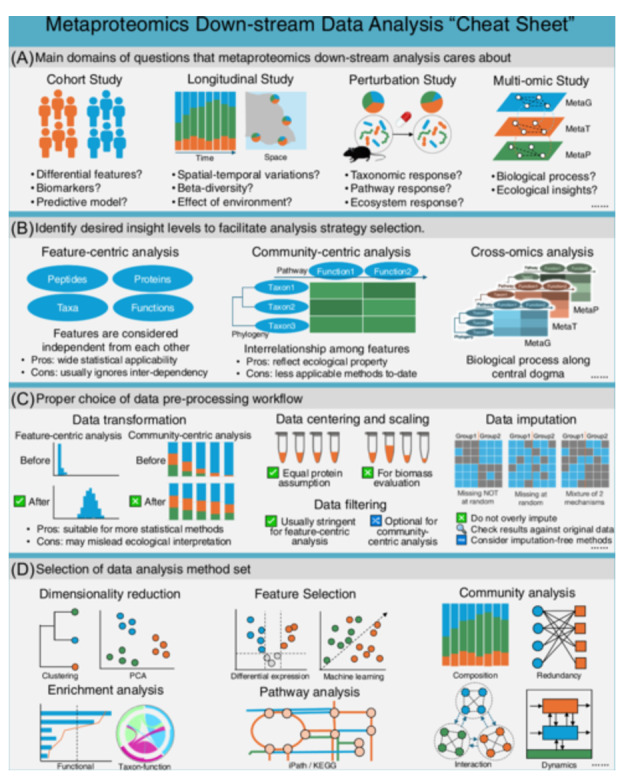
Metaproteomics downstream data analysis “cheat sheet.” (A) Main domains of questions that metaproteomics downstream analysis cares about. (B) Identify desired insight levels to facilitate analysis strategy selection. (C) Proper choice of data pre‐processing workflow. (D) Selection of data analysis method set.

A common question among researchers is how to determine the optimal approach for downstream processing of metaproteomic data. Unfortunately, there is no universal workflow that fits every scenario. This section aims to guide readers in constructing a tailored decision tree for analyzing metaproteomic data sets. In earlier sections, we detailed the generation of various metaproteomic data tables, including peptides, proteins, taxonomy, and functional attributes. The next step involves uncovering the underlying patterns and biological insights within these data sets through statistical analysis. Designing a robust statistical analysis pipeline for metaproteomics requires researchers to make several informed decisions, which are summarized in a “cheat sheet” in Figure 7.

#### Identifying relevant scientific questions

The foundation of any metaproteomics analysis begins with defining the key scientific question(s) of the study. Metaproteomics allows us to address a variety of research objectives. Below are some common examples of questions that can be explored (Figure 7A): (i) Cohort studies: What differential features distinguish healthy individuals from those with a disease? Are there potential biomarkers for specific conditions? (ii) Microbiome dynamics: How does the microbiome vary over space and time? Can beta diversity be observed at the functional ecological level? What is the impact of specific environmental factors on the microbiome? (iii) Perturbation study: How do microbial communities respond to external perturbations at the taxonomic, functional, and ecological levels? (iv) Multi‐omics study: What (holistic) insights can be gained by integrating metaproteomics with other omics approaches?

#### Selecting appropriate levels of analytical insights

Once the primary research questions are defined, the next step is to determine the level of insights required to address these questions (Figure 7B). This involves selecting between different analytical approaches tailored to the objectives of the study.

(i) Feature‐centric analysis

Feature‐based methods are the most commonly applied in metaproteomics. These analyses focus on identifying differential features, which are quantifiable variables that exhibit statistically significant differences between groups or conditions. Examples include specific peptides, proteins, taxonomic groups, or annotated functions that vary significantly under different experimental condition.

There are two key considerations that underpin feature‐centric analysis: (i) the assumption of standard statistical distributions, such as normality, to validate analytical methods, and (ii) the treatment of features as independent variables, enabling the use of widely‐applied statistical approaches like parametric or non‐parametric tests. By adhering to these principles, feature‐centric analyses enable robust identification of biologically meaningful differences across data sets.

(ii) Community‐centric analysis

Unlike feature‐centric analysis, community‐centric analysis considers the data set as a reflection of a living ecological community. Here, proteins are viewed not as isolated features but as components of interconnected networks, with functions linked through evolutionary relationships and taxonomic origins. For example, proteins from different taxa may exhibit functional redundancy, while ecological dynamics may influence functional and taxonomic interactions.

Due to these complex interactions, traditional statistical methods that assume feature independence may not be suitable. To address these challenges, novel ecological approaches have been developed in metaproteomics, inspired by advancements in metagenomics.

For example, metrics for functional redundancy utilize bipartite networks to link taxonomic and functional attributes, serving as indicators of community health and stability [43, 306]. Similarly, PhyloFunc, integrates phylogenetic composition into functional beta diversity analysis by incorporating functional distances at nodes of phylogenetic trees and applying a unifrac‐like weighting scheme [307]. This approach distinguishes whether functional changes result from compensation among closely related species or shifts between distantly related taxa, offering valuable insights into ecological dynamics.

(iii) Cross‐omics analysis

The metaproteome is intrinsically linked to other meta‐omes, making the integration of multiple omics data sets essential for a deeper understanding of microbiome systems ecology. Different meta‐omics approaches possess complementary strengths as they collectively capture variations along the central dogma of molecular biology (DNA → RNA → Protein), favoring a comprehensive understanding of biological processes and ecological interactions within microbiomes.

Despite the complementary nature of these data sets, most studies have traditionally analyzed meta‐omics using separate, stand‐alone workflows. However, recent advances in bioinformatics tools and platforms, such as Galaxy [308] and MOSCA [309], have facilitated the integration of these data sets, enabling more seamless and coherent analysis. Cross‐omics analysis can also provide an in‐depth view of the functional dynamics of community ecology.

In a recent study, metagenomics and metaproteomics were paired to assess whether certain proteins serve as niche proteins (proteins that contribute to the ecological role or niche that a microbial community occupies within its environment) or play essential metabolic roles within a community [310]. To achieve this, genome‐ and proteome‐level functional redundancy within the community were compared simultaneously. A larger discrepancy might indicate that certain genes are present but not expressed as proteins, suggesting a more specialized or niche role. Smaller discrepancies might indicate that the genes are actively translated into proteins, suggesting essential metabolic functions.

#### Data preprocessing strategies

After selecting appropriate levels of analytical insights, the first step in downstream analysis is data preprocessing. Common preprocessing steps include data filtering, data transformation, data imputation, and data scaling (Figure 7C). However, there is no universal approach for data preprocessing; the best strategy depends on the specific research questions under investigation.

(i) Data transformation

Common data transformations used in proteomics and metaproteomics include logarithmic transformations (e.g., log2 or log10) and square root transformations. However, not all scenarios are suitable for data transformation.

When to use data transformation: Transformation is recommended when achieving near‐normality in the data is necessary. For feature‐level analyses, log transformation of peak intensities can make the data approximate a normal distribution. Normal distributions are crucial for many commonly applied metaproteomic feature selection methods, such as linear models, empirical Bayes, univariate *t*‐tests, partial least squares discriminant analysis (PLS‐DA), and orthogonal partial least squares discriminant analysis (OPLS‐DA). If the data are not normally distributed, alternative non‐parametric methods may be considered to meet the assumptions of the chosen analysis.

When not to use data transformation: Transformation should be avoided when reflecting protein abundance. For example, volcano plots, often used for identifying differential features, plot statistical significance (−log_10_(*p*‐value)) against fold change (log_2_ fold change). While fold change values are log‐transformed for visualization purposes, the original fold change data should remain untransformed during statistical analyses or comparisons. Additionally, in community‐level analyses, log transformation can obscure protein biomass information, which is essential for estimating taxonomic and functional compositions. Protein intensities or PSM counts can serve as reliable measures of protein biomass contributions by taxa [167]. Therefore, composition‐based analyses, such as alpha and beta diversity or functional redundancy assessments, should use untransformed data.

(ii) Data centering and scaling

In standard metaproteomics workflows, an equal amount of protein is typically extracted from each sample, digested, and loaded into the mass spectrometer to ensure consistency and comparability. However, in specific cases, metaproteomics may quantify overall protein biomass responses based on the total protein biomass in a given system volume rather than standardizing based on protein content [42]. In such cases, centering and scaling are not recommended. Alternative normalization techniques, such as total spectral count normalization or median normalization, may be more appropriate for these scenarios.

(iii) Data filtering

Filtering the data set typically helps remove noise, irrelevant features, or outliers. The application of data filtering should be tailored to the specific context of the study.

For feature‐centric analysis, stringent data filtering is crucial, particularly when identifying biomarkers. This process involves setting a higher threshold for protein presence across samples to ensure that identified biomarkers are consistently detected in the majority of subjects. By requiring proteins to be present in a large percentage of samples (e.g., 70%–90%), researchers can improve the reliability and relevance of the identified biomarkers. This consistency is critical for validating potential biomarkers, as it reduces the likelihood of identifying false positives. Data filtering is also typically stringent for other types of feature‐centric analysis to ensure the validity of statistical hypotheses. However, the threshold and method of filtering (e.g., by the whole data set or by group) must be properly applied to prevent over‐filtering, which could remove features that are truly missing in specific subgroups.

For community‐centric data analysis, filtering is optional, with less stringent thresholds allowing for a more comprehensive view of community dynamics. While some filtering helps remove obvious noise, it is applied more flexibly than in feature‐centric analysis. For example, unfiltered taxon‐specific functional data can provide a better review of the degree distribution of functions in a microbiome [306].

(iv) Data imputation

In a metaproteomic data set, missingness often arises from two simultaneous mechanisms. First, the diversity and sparse nature of the metaproteome lead to a significant proportion of true missing proteins (missing not at random). Second, the inherent depth limitation of current common metaproteomic techniques results in highly sparse detection of low‐abundance proteins across samples (missing at random) [311].

Data imputation is the step that requires the most caution. Improper selection of the data imputation approach can induce false positives. When a large proportion (e.g., > 50%) of a feature is missing, excessive imputation can lead to the creation of artificial values that do not reflect the true biological scenario and, in some cases, can further lead to false positives. If the imputation method does not accurately reflect the nature of the missing data, it can introduce bias, particularly if the data contains a mixture of both missingness mechanisms. If features have been selected through a statistical test following data imputation, it is recommended to always revisit the un‐imputed data to double‐check if the feature‐level difference is true before drawing solid conclusions.

Alternatively, a univariate selection method has been which combines a test of association between missingness and classes with a test for the difference in observed intensities between classes. This method provides a robust alternative for handling missing data without relying on imputation [311].

Notably, data imputation is essential for feature selection analysis, whereas for community‐level approaches, it is typically unnecessary, for reasons similar to those explained above.

#### Choosing data analysis methods

After a thorough understanding and careful selection of preprocessing steps, the final step in downstream data analysis is the selection of appropriate methods (Figure 7D). This stage presents significant opportunities for deriving diverse insights from the data set and is often the most engaging and time‐consuming phase, allowing researchers to explore the data and uncover meaningful biological or ecological patterns and conclusions. These strategies typically include, but are not limited to:

Dimensionality reduction: Dimensionality reduction methods are commonly used to uncover underlying patterns or structures within the data set and to assess similarities between samples. Unsupervised methods such as PCA, t‐distributed Stochastic Neighbor Embedding (t‐SNE), hierarchical clustering, and k‐means clustering are frequently applied. Supervised methods, such as Partial Least Squares Discriminant Analysis (PLS‐DA), are also widely utilized. Dimensionality reduction is applicable not only to peptide, protein, taxonomic, and functional tables but also at the MS1 level, especially when the primary goal is to reveal patterns between samples [312].

Enrichment analysis: Enrichment analysis determines whether a subset of selected features is significantly over‐represented compared to a background database. While enrichment analysis can be implemented using programming languages such as R, iMetaShiny [300] offers interactive functionality for taxonomic and functional enrichment analysis of protein IDs or Clusters of Orthologous Groups (COG) IDs. However, protein ID‐based enrichment analysis is currently restricted to human gut metaproteome analysis using the Integrated Gene Catalog (IGC) database.

Feature Selection: Several online tools, such as MetaFS [313], MetaQuantome [314], MetaX [315], iMetaShiny [300], and stand‐alone tools, such as Meta4P [316], have been developed to facilitate feature‐based metaproteomic data analysis without requiring extensive programming expertise.

Pathway analysis: Pathway analysis is typically employed to gain an overview of detected functions or to compare differentially expressed or enriched pathways across groups. The most commonly used tools for pathway analysis include KEGG mapper [286] and iPath [317]. More recently, PathwayPilot was developed to easily compare functions at the KEGG pathway level, either between selected taxa within a single sample or across different samples, by leveraging Enzyme Commission numbers (EC numbers) to identify active enzymes as proxies for metabolites linked to KEGG maps, thereby facilitating investigations into functions associated with specific conditions while allowing targeted analysis of selected species [318].

Community analysis: Beyond feature‐driven analysis, community‐level analysis focuses on viewing the entire metaproteome as a dynamic system. Such analyses may include inferring community composition, alpha diversity, beta diversity, and functional redundancy using metaproteomic data.

## A COLLABORATIVE EFFORT: WRITING A COMPREHENSIVE REVIEW WITH MEMBERS OF THE METAPROTEOMICS INITIATIVE

The Metaproteomics Initiative is an international community dedicated to advancing the field of metaproteomics within microbiome research. Supported by the European Proteomics Association (EuPA) and the Human Proteome Organization (HUPO), and in collaboration with the European life sciences infrastructure ELIXIR [319], this initiative serves as a central hub for researchers to disseminate advancements, share methodologies, and establish standards across the metaproteomics community.

This Initiative aims to facilitate communication between experts and newcomers, standardize practices, and accelerate developments in metaproteomic methodologies. Its primary mission is to be the go‐to resource for metaproteomics fundamentals, advancements, and applications, fostering a collaborative network to drive forward experimental and bioinformatic methodologies.

The Metaproteomics Initiative supports on three pillars: (i) Communication and Collaboration: This pillar focuses on sharing field advancements, organizing benchmark studies like CAMPI, and hosting the International Metaproteomics Symposium (IMS); (ii) Education & Outreach: The initiative educates the broader microbiome community through accessible resources, including webinars and workshops, and facilitates expert interactions; and (iii) Standardization: Efforts are directed toward developing robust (meta)data standards, promoting FAIR data principles to ensure accessible and reusable research outputs.

As part of our commitment to Education & Outreach, we created this review to make metaproteomics accessible to a broad audience. To ensure a thorough and well‐rounded perspective, we first invited experts in various areas to draft individual sections. These drafts were then reviewed internally, where initial feedback helped refine each section. Once authors made adjustments, the document went through additional rounds, allowing all contributors to share insights and address any remaining comments. The specific contributions of each author are documented in the Supplementary Information.

In the next step, we brought in microbiome researchers who were new to metaproteomics to review the manuscript, helping us ensure it was clear and approachable to those outside the field. With their feedback integrated, all co‐authors—including section authors and both expert and novice reviewers—had a final opportunity to review the work. This collaborative approach allowed us to prepare a comprehensive, accessible resource, which we shared as a preprint before journal submission.

## CONCLUSION

This *Microbiologist's Guide to Metaproteomics* is designed for microbiome researchers starting in metaproteomics, offering a practical introduction to reduce barriers to entry. It covers the essentials of metaproteomics, including experimental design, sample preparation, mass spectrometry data acquisition, peptide identification, protein inference, taxonomic and functional analysis, and basic statistical methods. The guide provides the foundational knowledge needed to apply metaproteomic technologies in microbiology and microbiome studies. Metaproteomics is a rapidly evolving field with unresolved technical challenges and unexplored areas. This guide focuses on foundational concepts rather than providing exhaustive coverage. To address these challenges, the Metaproteomics Initiative launched the “Critical Assessment of Metaproteome Investigations (CAMPI)” series, which facilitates multi‐laboratory collaborations to compare and improve workflows, including sample preparation, mass spectrometry methods, and bioinformatics. Looking ahead, the next decade promises remarkable advancements in mass spectrometry, with continually improving performance deepening the coverage of metaproteomic analysis. These advancements, coupled with ongoing and future enhancements in wet‐lab protocols, strategies, and bioinformatic tools, will further propel the field. Collaborative efforts, such as the CAMPI series of the Metaproteomics Initiative, underscore the power of cooperation in driving metaproteomic progress. These developments, supported by input from microbiome researchers, will help deepen our understanding of microbiomes and their functions in diverse ecosystems.

## AUTHOR CONTRIBUTIONS

**Tim Van Den Bossche**: Writing—original draft; writing—review and editing; project administration. **Jean Armengaud**: Writing—original draft; writing—review and editing. **Dirk Benndorf**: Writing—original draft; writing—review and editing. **Jose Alfredo Blakeley‐Ruiz**: Writing—original draft; writing—review and editing. **Madita Brauer**: Writing—review and editing. **Kai Cheng**: Writing—original draft; writing—review and editing. **Marybeth Creskey**: Writing—original draft; writing—review and editing. **Daniel Figeys**: Writing—original draft; writing—review and editing. **Lucia Grenga**: Writing—original draft; writing—review and editing. **Timothy J. Griffin**: Writing—original draft; writing—review and editing. **Céline Henry**: Writing—review and editing. **Robert L. Hettich**: Writing—original draft; writing—review and editing. **Tanja Holstein**: Writing—original draft; writing—review and editing. **Pratik D. Jagtap**: Writing—original draft; writing—review and editing. **Nico Jehmlich**: Writing—original draft; writing—review and editing. **Jonghyun Kim**: Writing—review and editing. **Manuel Kleiner**: Writing—original draft; writing—review and editing. **Benoit J. Kunath**: Writing—original draft; writing—review and editing. **Xuxa Malliet**: Writing—review and editing. **Lennart Martens**: Writing—original draft; writing—review and editing. **Subina Mehta**: Writing—original draft; writing—review and editing. **Bart Mesuere**: Writing—original draft; writing—review and editing. **Zhibin Ning**: Writing—original draft; writing—review and editing. **Alessandro Tanca**: Writing—original draft; writing—review and editing. **Sergio Uzzau**: Writing—original draft; writing—review and editing. **Pieter Verschaffelt**: Writing—original draft; writing—review and editing. **Jing Wang**: Writing—review and editing. **Paul Wilmes**: Writing—original draft; writing—review and editing. **Xu Zhang**: Writing—original draft; writing—review and editing. **Xin Zhang**: Writing—review and editing. **Leyuan Li**: Writing—original draft; writing—review and editing; visualization; project administration.

## CONFLICT OF INTEREST STATEMENT

Daniel Figeys is a Cofounder of MedBiome inc.

## ETHICS STATEMENT

No animals or humans were involved in this study.

## Supporting information

Supporting information

## Data Availability

Data sharing not applicable to this article as no data sets were generated or analyzed during the current study. This manuscript did not generate or use any data sets requiring data availability. Supplementary materials (graphical abstract, slides, videos, Chinese translated version and update materials) may be found in the online DOI or iMeta Science http://www.imeta.science/.
