# Supplementary material for: The microbiologist's guide to metaproteomics
Source: Imeta. 2025 May 6;4(3):e70031. doi: 10.1002/imt2.70031 (PMC12130581; doi:10.1002/imt2.70031)
Supplement: Supplementary file 1 — Supporting information [file IMT2-4-e70031-s001.docx]

**AUTHOR CONTRIBUTIONS BY SECTIONS**

This review is a collaborative effort led by Tim Van Den Bossche and Leyuan Li, and overseen by the Scientific Committee of the Metaproteomics Initiative, who provided overall guidance. Each section was contributed by nominated authors and internal reviewers as follows: Section 1: Why metaproteomics? was written by Robert Hettich, Jean Armengaud, Dirk Benndorf, Paul Wilmes, and Madita Brauer, and reviewed by Daniel Figeys. Section 2: Basics of proteomics was written by Zhibin Ning and Daniel Figeys, and reviewed by Leyuan Li. Section 3: Experimental methods in metaproteoimcs includes several subsections: 3.1 Experiment Design was written by Lucia Grenga and Jean Armengaud, reviewed by Céline Henry and Leyuan Li; 3.2 Sample collection, preservation, and storage prior to preprocessing, where 3.2.1 Sample collection and preservation was written by Sergio Uzzau and Alessandro Tanca, and 3.2.2 Storage conditions to maintain sample integrity was written by Lucia Grenga and Jean Armengaud, both reviewed by Céline Henry and Leyuan Li; 3.3 Sample preprocessing was written by Lucia Grenga and Jean Armengaud, reviewed by Céline Henry; 3.4 Protein sample preparation: from extraction to digestion was written by Nico Jehmlich, reviewed by Xu Zhang and Céline Henry; 3.5 Separation and fractionation techniques was written by Xu Zhang and Marybeth Creskey, reviewed by Céline Henry; 3.6 Automation was written by Leyuan Li, reviewed by Sergio Uzzau and Alessandro Tanca; 3.7 Mass spectrometry data acquisition methods was written by Zhibin Ning and Daniel Figeys, reviewed by Jean Armengaud and Céline Henry. Section 4: Computational analysis of metaproteomics data includes several subsections: 4.1.1 Peptide identification with proteomics search engines was written by Pratik Jagtap, Subina Mehta, and Timothy Griffin, reviewed by Tanja Holstein and Kai Cheng; 4.1.2 Database construction or selection was written by Paul Wilmes and Benoit Kunath, reviewed by Jose Alfredo Blakely-Ruiz; 4.1.3 PSM FDR control, by Tim Van Den Bossche and Lennart Martens, reviewed by Tanja Holstein; 4.1.4 Protein inference was written by Tim Van Den Bossche, reviewed by Tanja Holstein; 4.1.5 Protein quantification was written by Jose Alfredo Blakely-Ruiz and Manuel Kleiner, reviewed by Tanja Holstein and Kai Cheng; 4.1.6 DIA data analysis was written by Pratik Jagtap, reviewed by Tanja Holstein and Kai Cheng; 4.2: Taxonomic and functional Analysis was written by Pieter Verschaffelt and Bart Mesuere, reviewed by Tanja Holstein and Tim Van Den Bossche; 4.3 Downstream statistics was written by Leyuan Li, reviewed by Tanja Holstein and Lucia Grenga. Section 5: A collaborative effort: writing a comprehensive review with members of the Metaproteomics Initiative was written by Tim Van Den Bossche, and reviewed by Leyuan Li. We invited Madita Brauer, Xuxa Malliet, Jing Wang, Xin Zhang, Jong Kim to review the manuscript to ensure its accessibility. All figures were artistically designed by Leyuan Li based on author drafts. To homogenize the text, ensure consistency and avoid redundancy across sections, all sections were rewritten by Tim Van Den Bossche. All authors commented and approved the final version of the manuscript.
